# Supplementary material for: Novel Derivatives of Quinoxaline-2-carboxylic Acid 1,4-Dioxides as Antimycobacterial Agents: Mechanistic Studies and Therapeutic Potential
Source: Pharmaceuticals (Basel). 2023 Nov 6;16(11):1565. doi: 10.3390/ph16111565 (PMC10675252; doi:10.3390/ph16111565)
Supplement: Supplementary file 1 [file pharmaceuticals-16-01565-s001.zip › pharmaceuticals-2628689-supplementary.pdf]

Supplementary Materials

Legends to Figures and Tables

Figure S1-S16. ^{1}H and ^{13}C NMR spectra of the compounds **4-9**. **S2**

Figure S17-S20. Copies of HRMS ESI spectra of the compounds **4-7**. **S10**

Figure S21-S24. Copies of HPLC spectra of the compounds **4-7**. **S14**

Figure S25-S28. Copies of IR spectra of the compounds **4-7**. **S18**

Figure S29-S38. Copies of 2D NMR spectra of **4** and **7**. **S22**

Table S1. Increments (I^C) for the ^{13}C chemical shift differences for the piperazine group for regioisomers **4** and **7** **S32**

Table S2. Stability of aqueous solutions of derivatives **4-7** **S33**

Figure S39. Nucleotide sequence of *MSMEG_4883*. **S34**

Table S3. Unique SNPs in quinoxaline-resistant *M. smegmatis* mutants **S35**

Table S4. Results of Dunnett's multiple comparison test for *in vivo* testing of compound **4**. **S41**

Figure S40. Results of MTT test of compounds on human fibroblasts. **S42**

Copies of NMR Spectra

Figure S1. Copy of ^{1}H NMR spectrum of the derivative **2**.

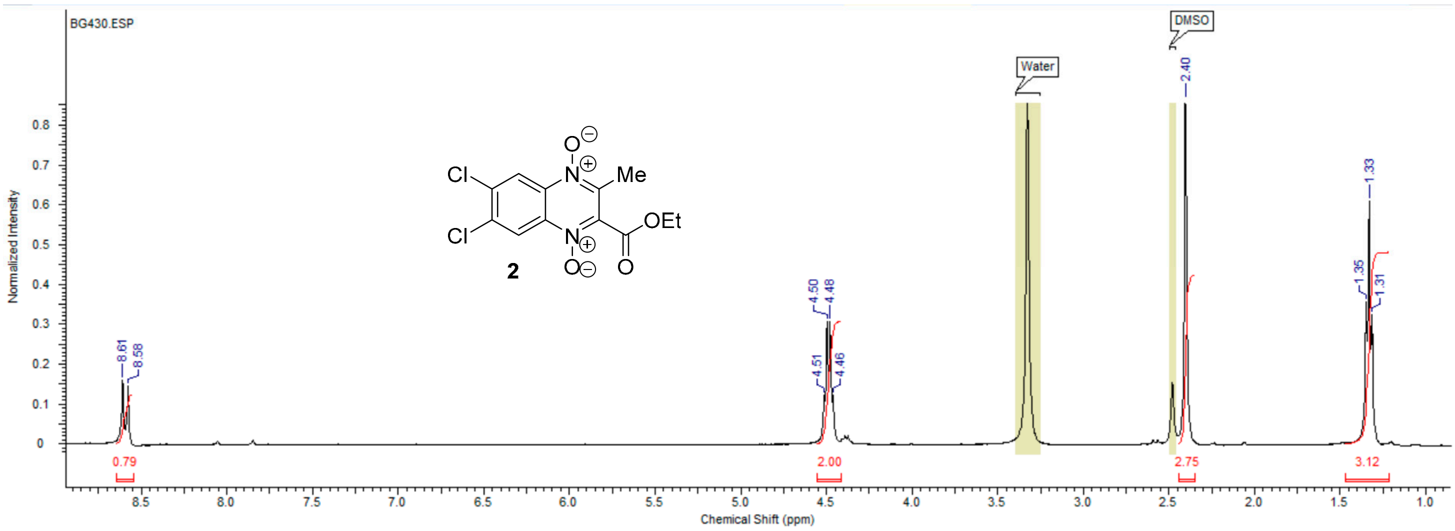

Figure S2. Copy of ^{13}C NMR spectrum of the derivative **2**.

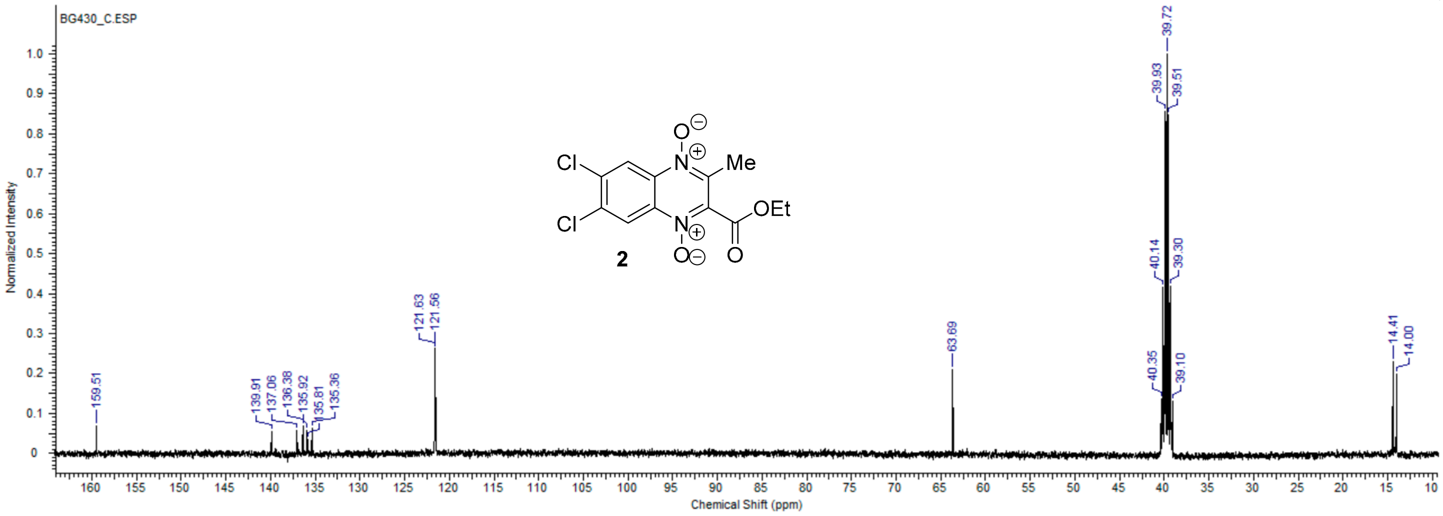

Figure S3. Copy of ^{1}H NMR spectrum of the derivative **3**.

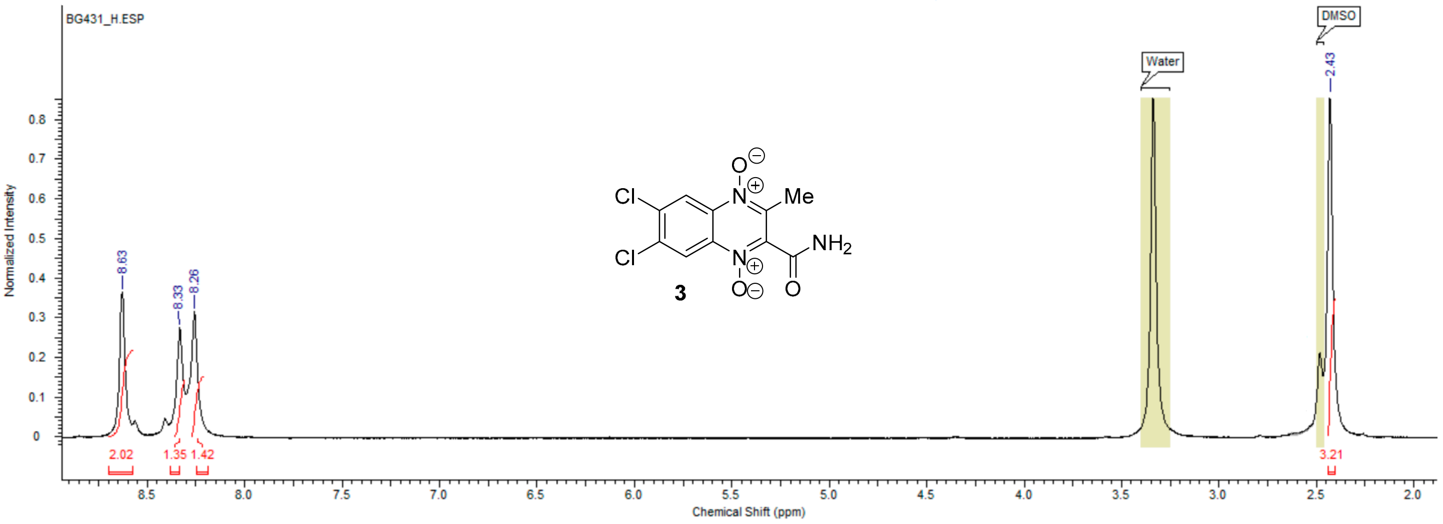

Figure S4. Copy of ^{13}C NMR spectrum of the derivative **3**.

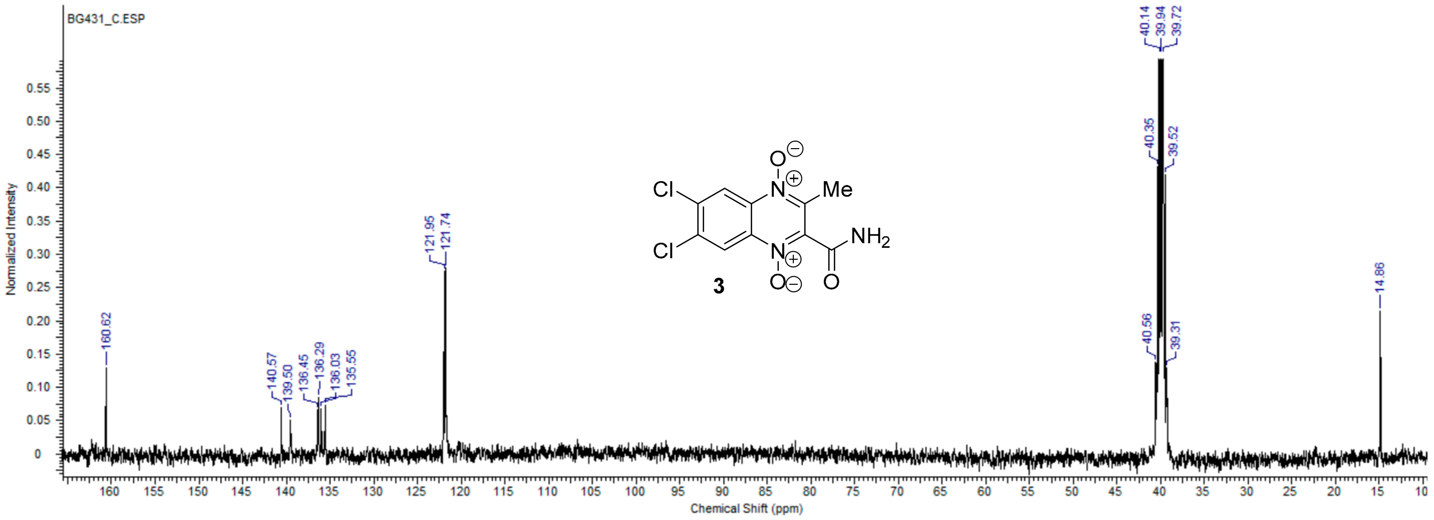

Figure S5. Copy of ^{1}H NMR spectrum of the derivative **4**.

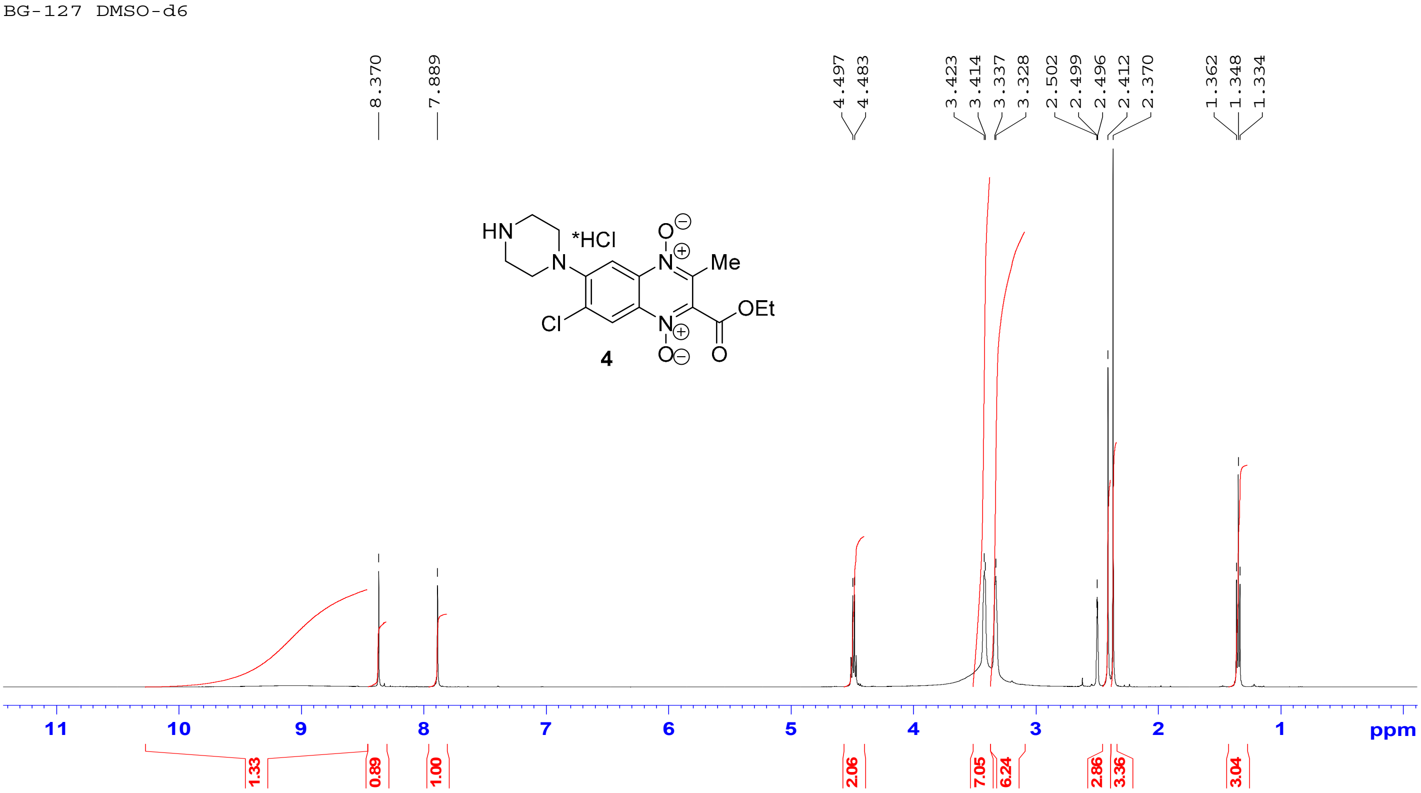

Figure S6. Copy of ^{13}C NMR spectrum of the derivative **4**.

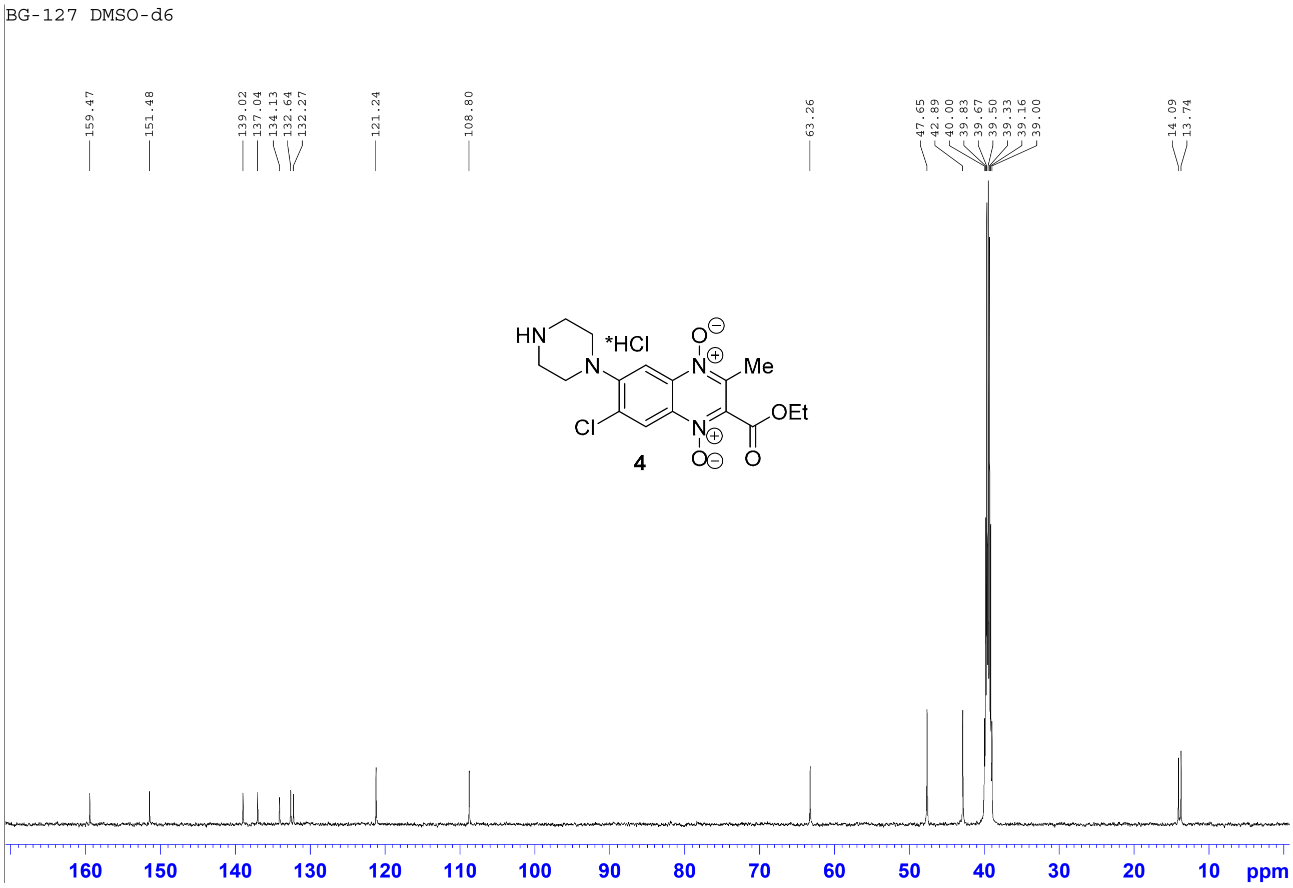

Figure S7. Copy of ^{1}H NMR spectrum of the derivative **5**.

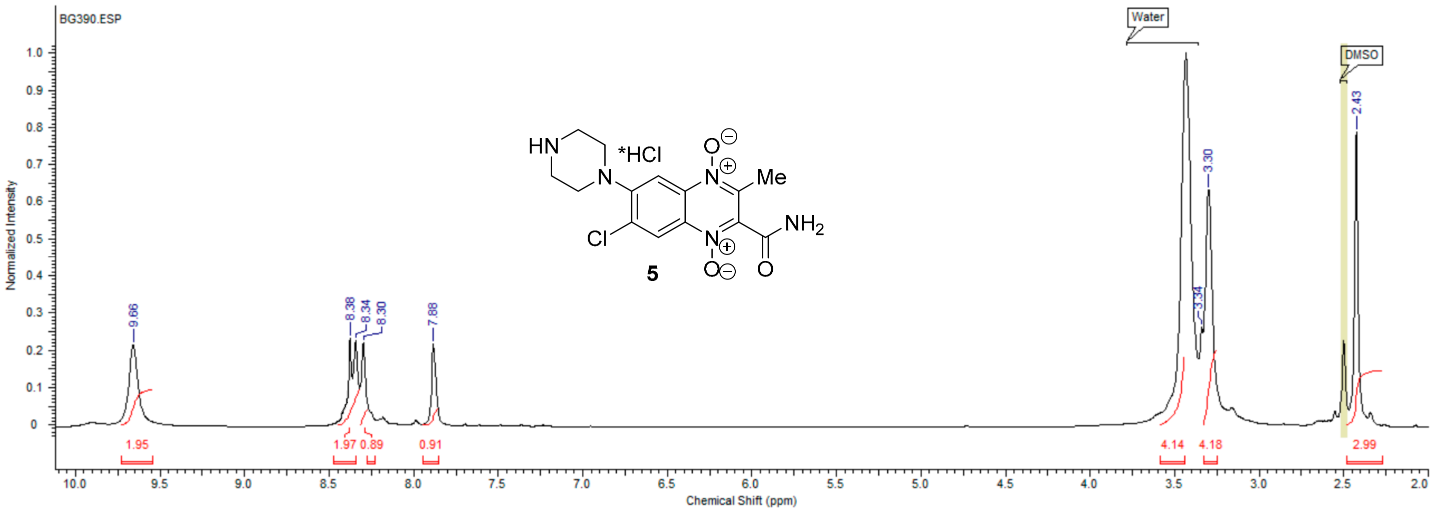

Figure S8. Copy of ^{13}C NMR spectrum of the derivative **5**.

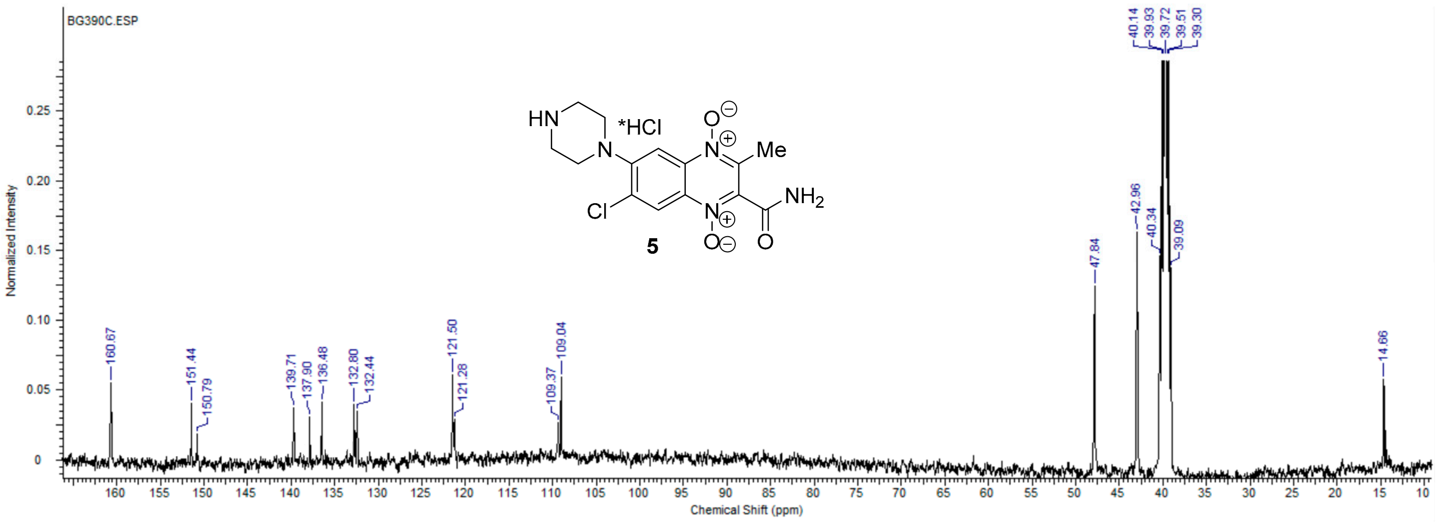

Figure S9. Copy of ^{1}H NMR spectrum of the derivative **6**.

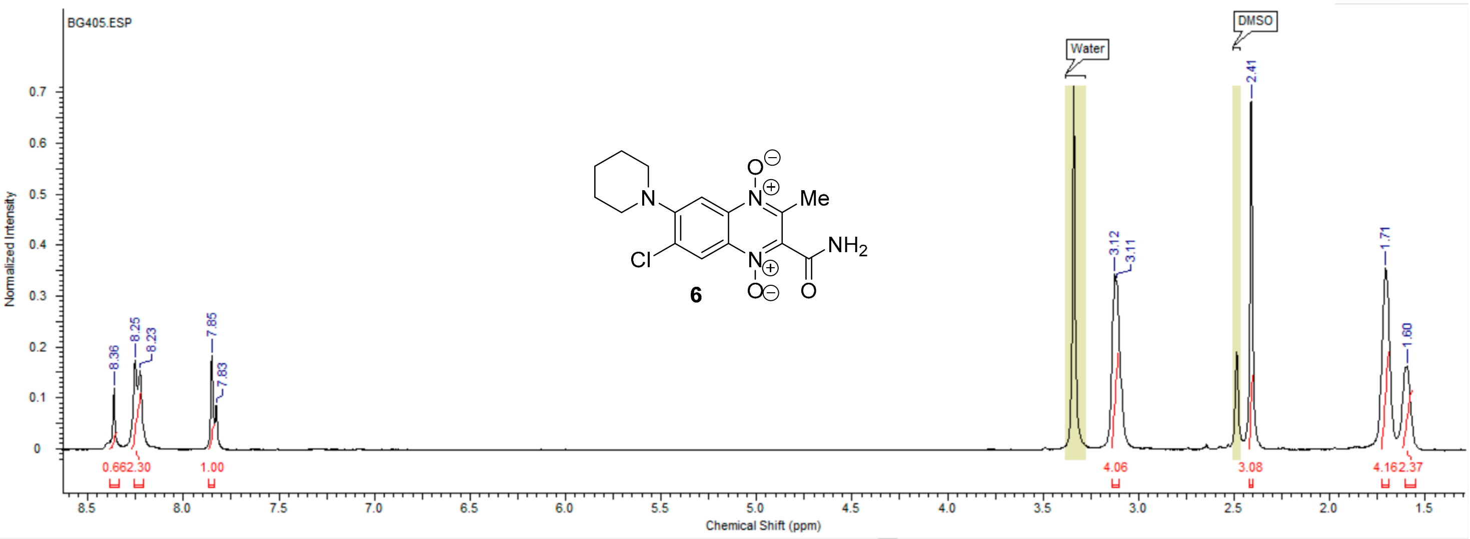

Figure S10. Copy of ^{13}C NMR spectrum of the derivative **6**.

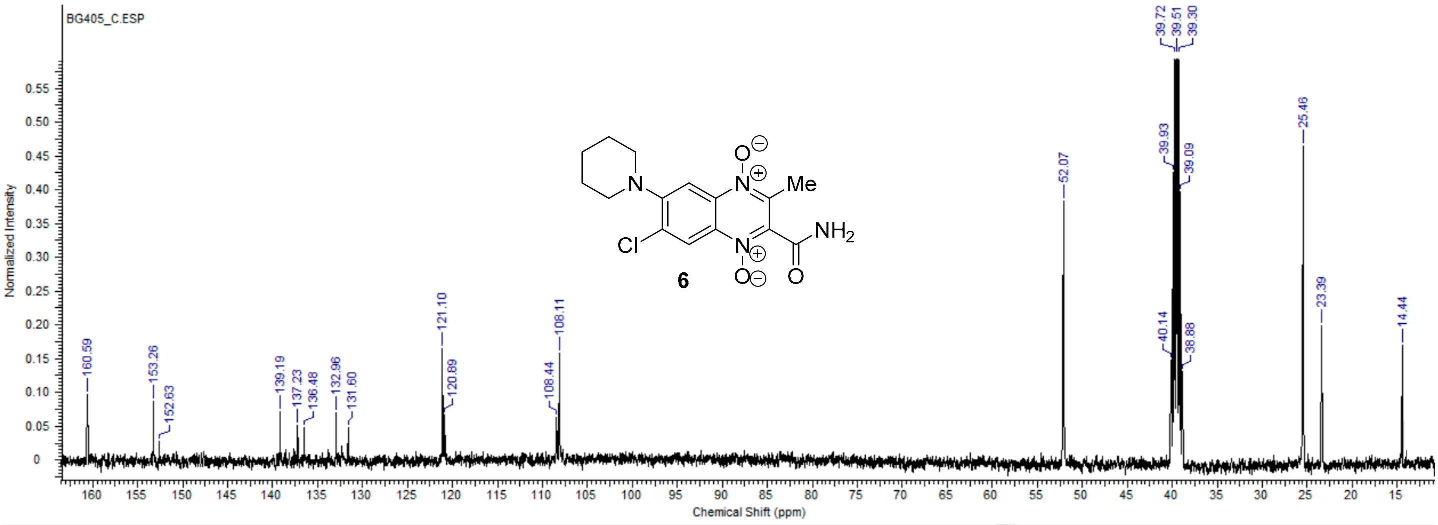

Figure S11. Copy of ^{1}H NMR spectrum of the derivative **7**.

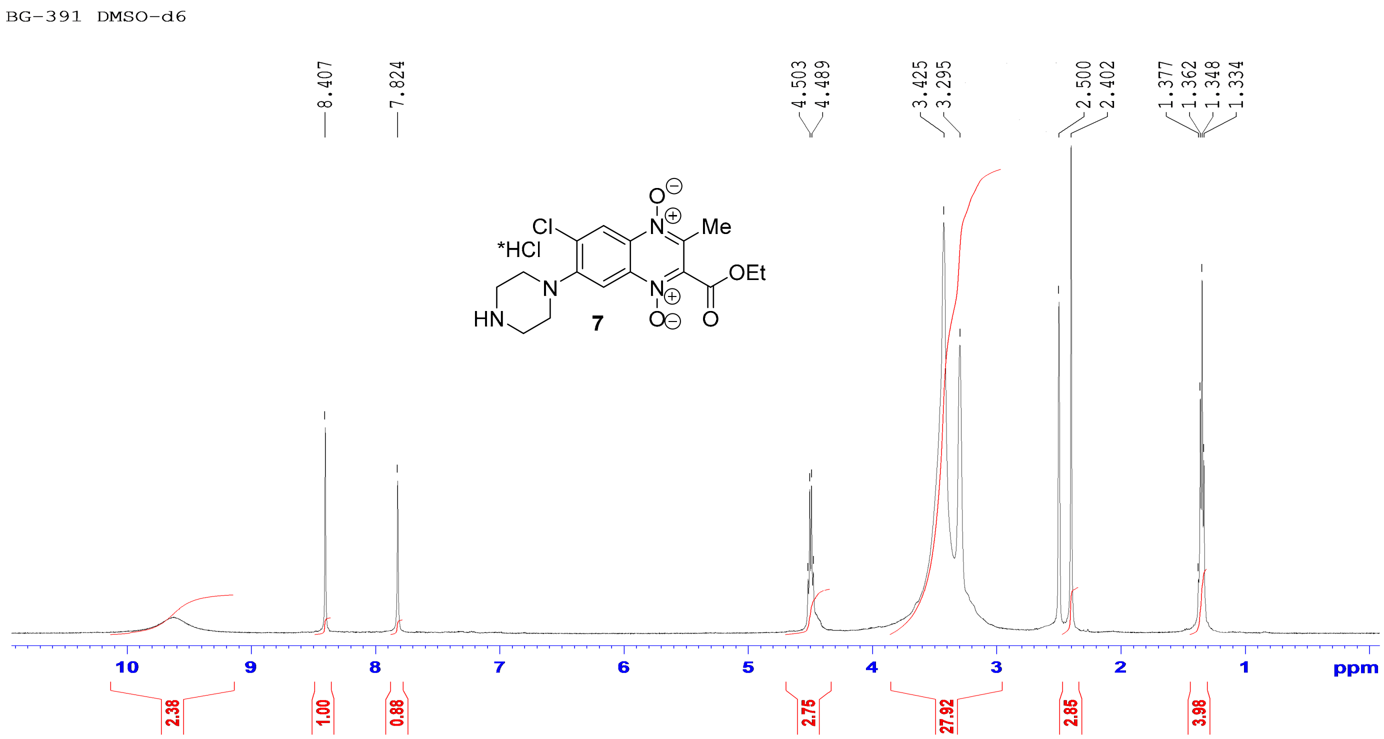

Figure S12. Copy of ^{13}C NMR spectrum of the derivative **7**.

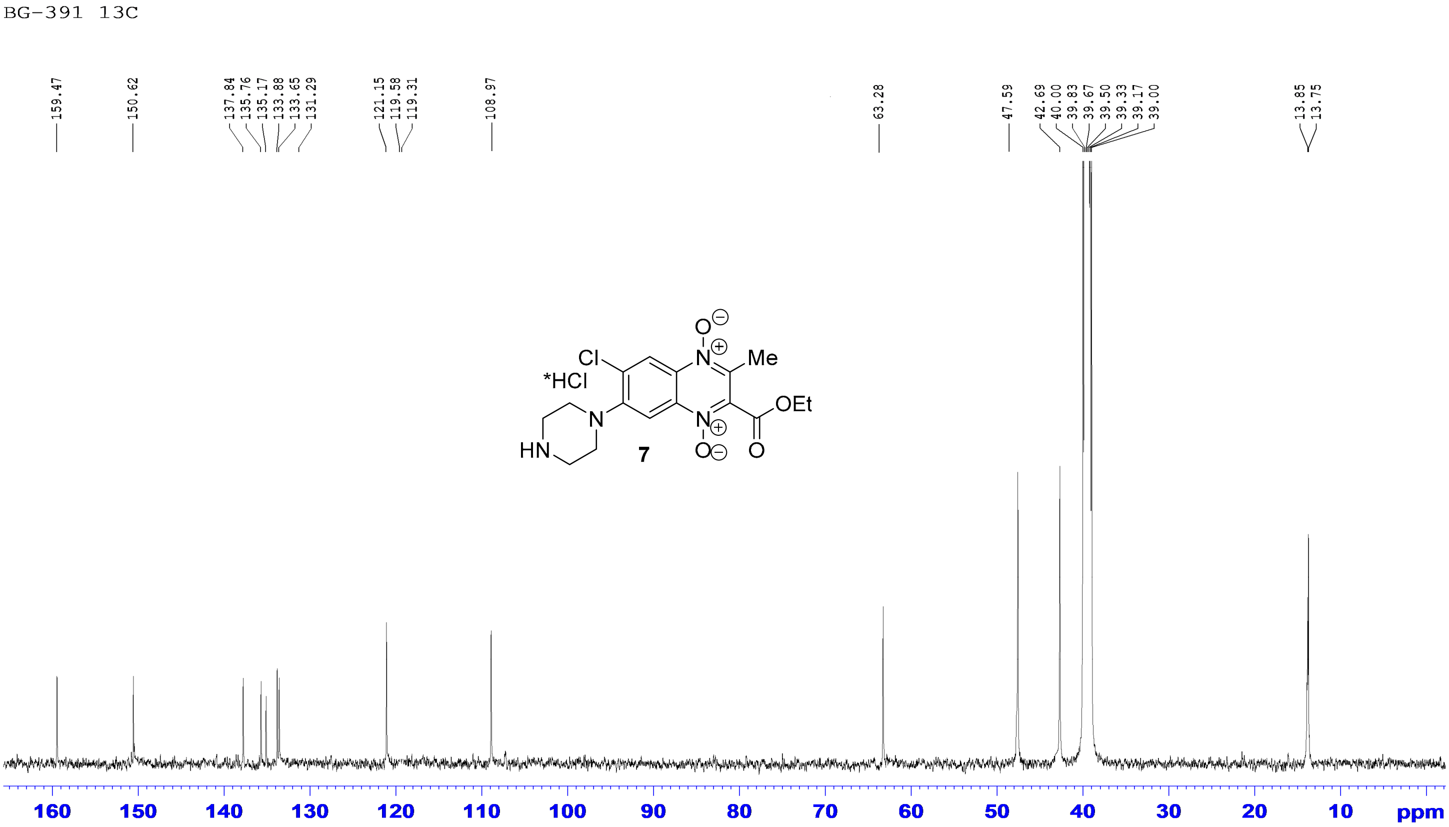

Figure S13. Copy of ^{1}H NMR spectrum of the derivative **8**.

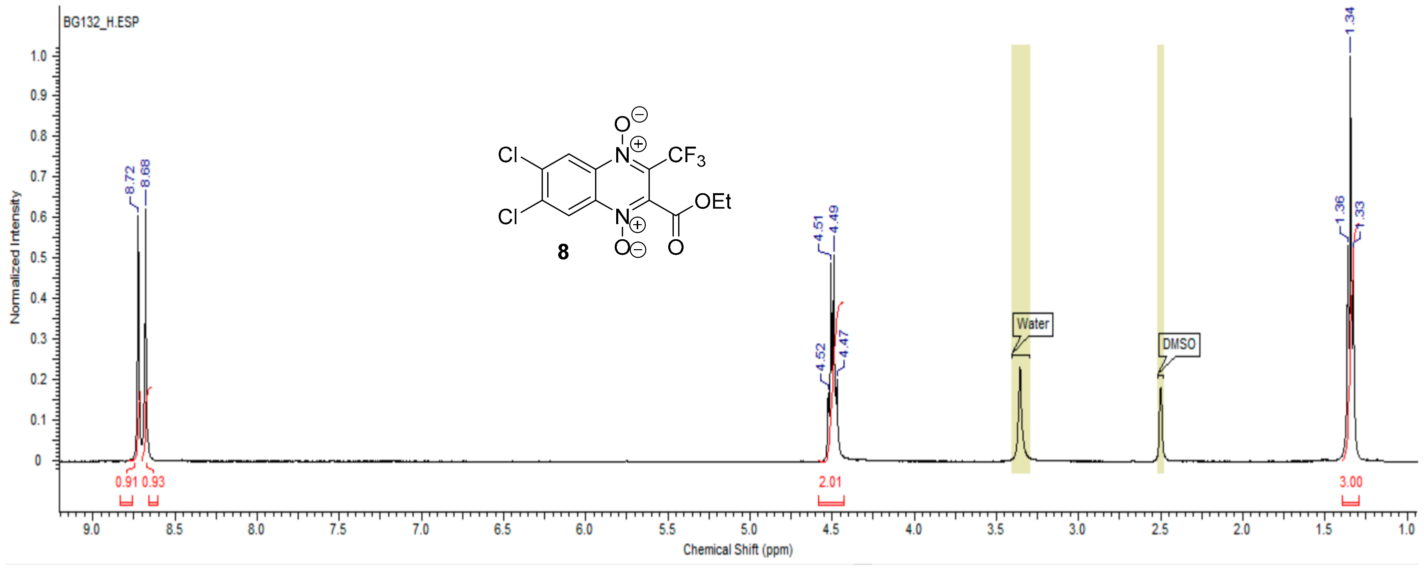

Figure S14. Copy of ^{13}C NMR spectrum of the derivative **8**.

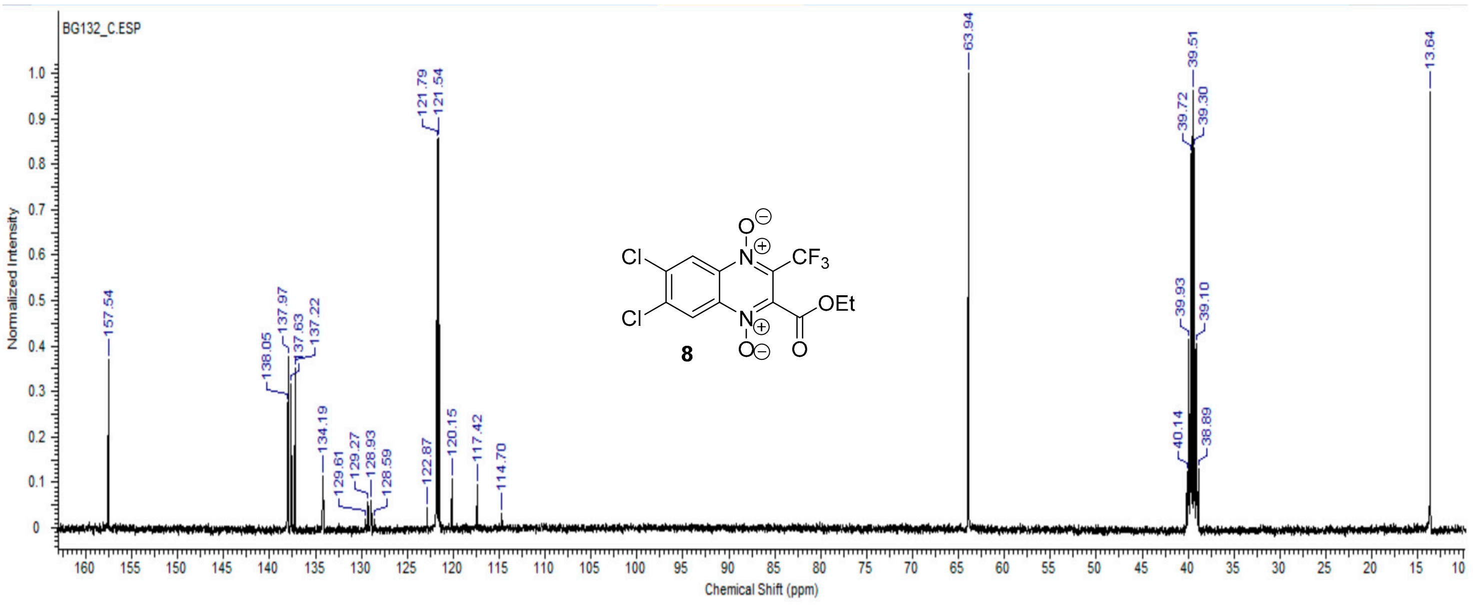

Figure S15. Copy of ^{1}H NMR spectrum of the 2-ethoxycarbonyl-3-methylquinoxaline 1,4-dioxide.

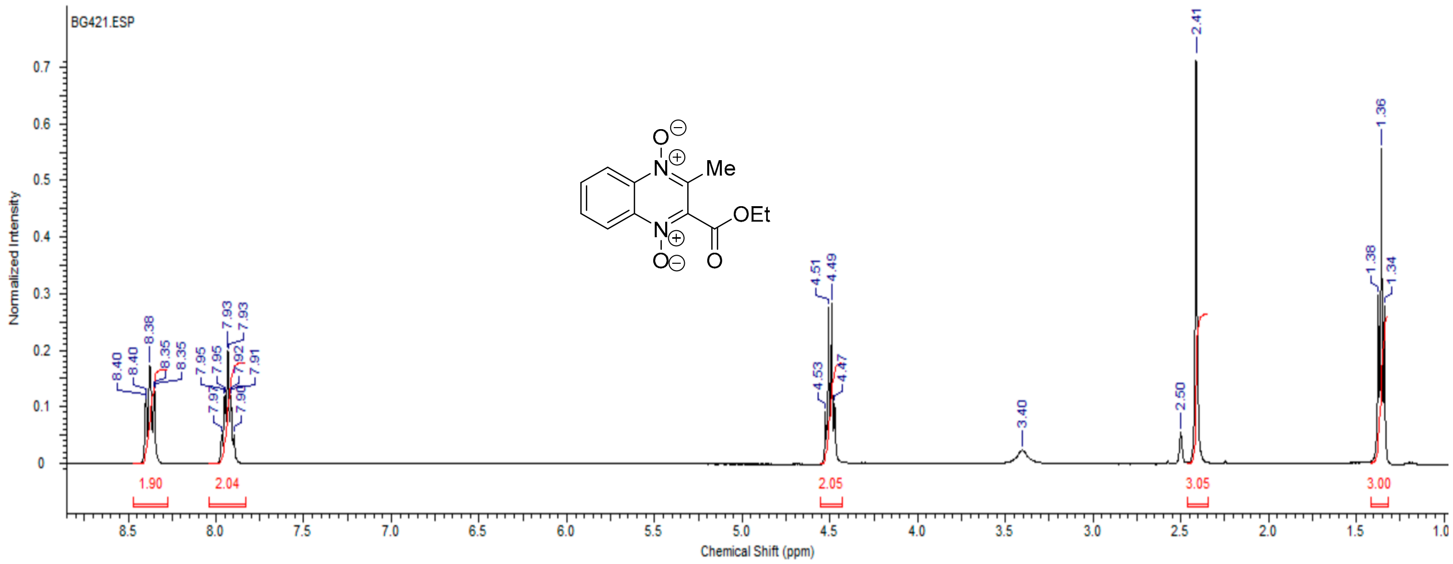

Figure S16. Copy of ^{13}C NMR spectrum of the derivative the 2-ethoxycarbonyl-3-methylquinoxaline 1,4-dioxide.

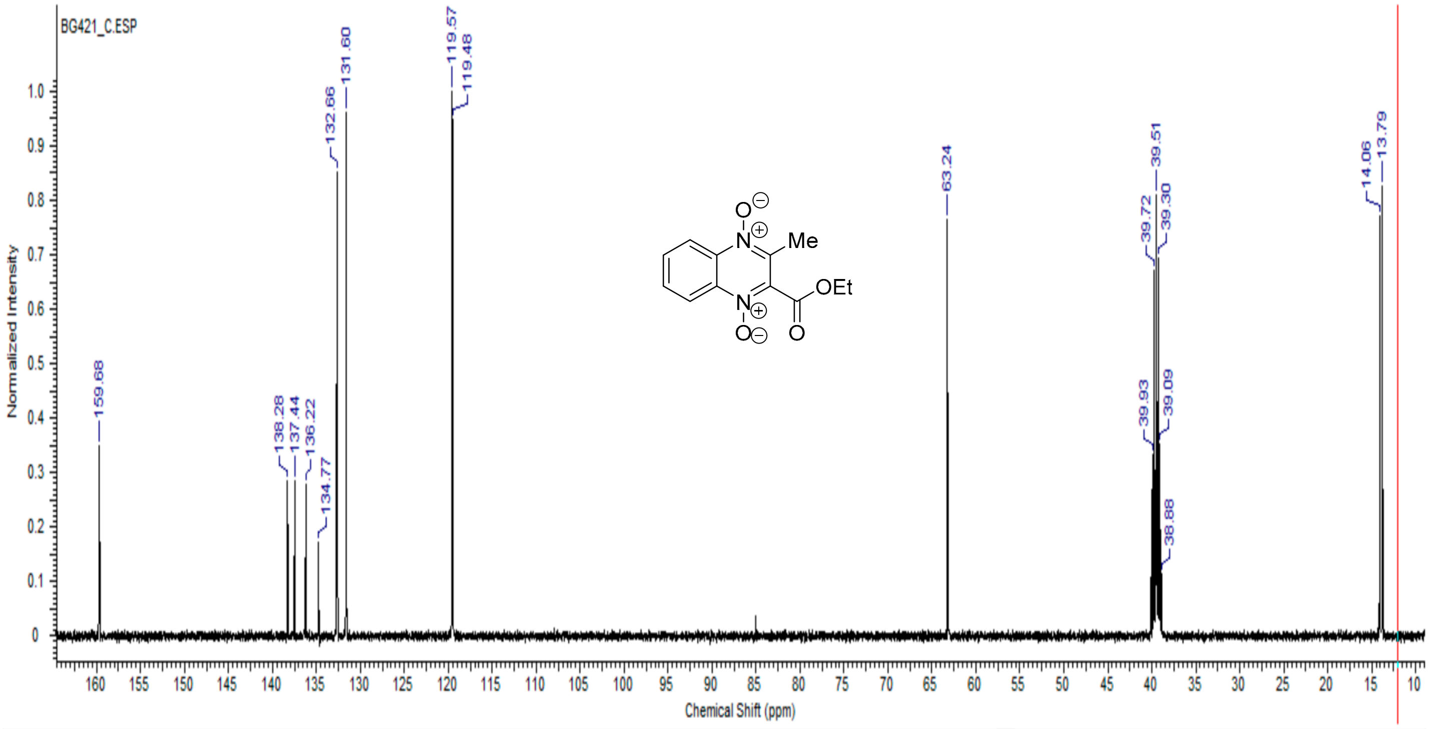

Copies of HRMS ESI Analysis

Figure S17. Copy of HRMS ESI analysis of the derivatives **4**.

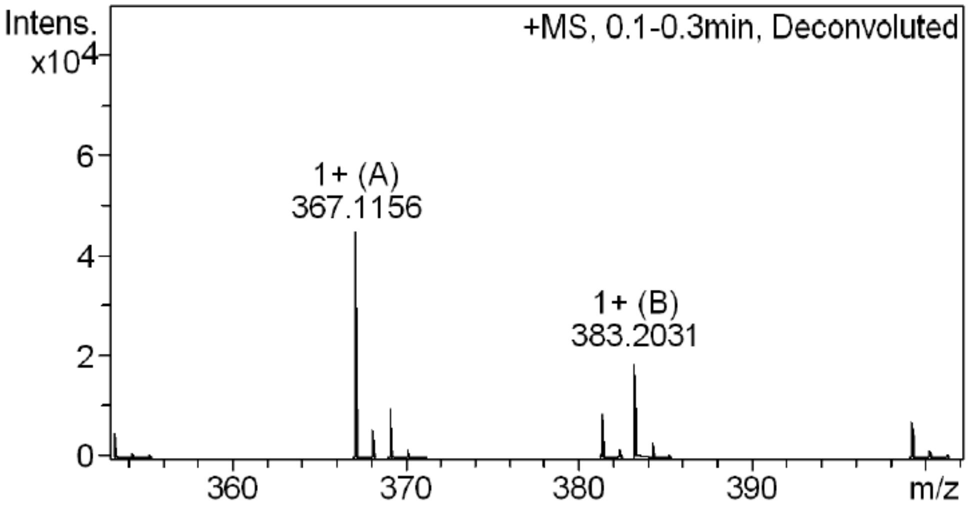

#	m/z	Res.	S/N	I	I %
1	353.2651			4980	10.9
2	367.1156			45857	100.0
3	369.1132			10487	22.9
4	381.2963			9106	19.9
5	383.2031			20309	44.3
6	399.1762			7620	16.6

Figure S18. Copy of HRMS ESI analysis of the derivatives **5**.

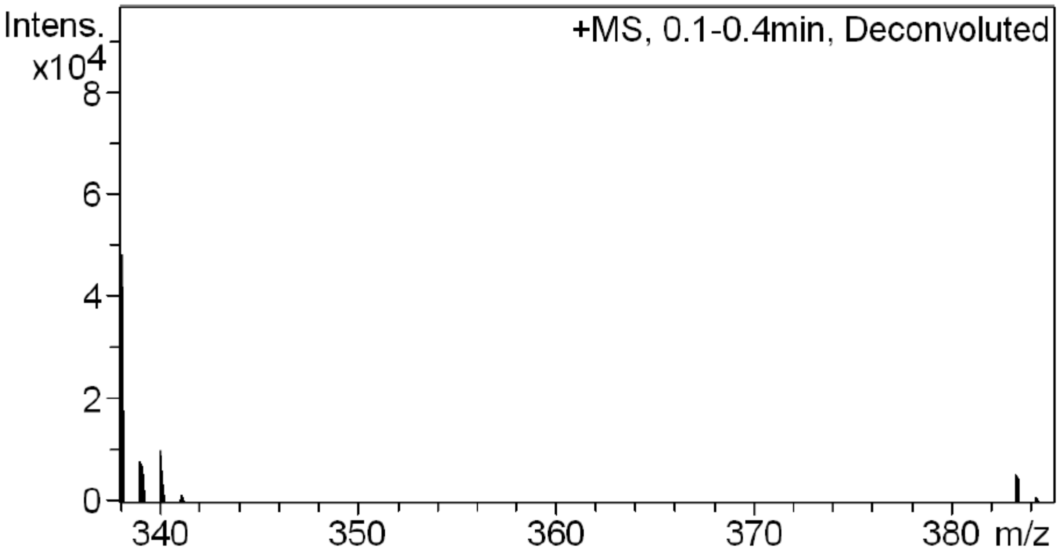

#	m/z	Res.	S/N	I	I %
1	338.1001			49185	100.0
2	340.0984			10435	21.2
3	383.2033			5443	11.1

Figure S19. Copy of HRMS ESI analysis of the derivatives **6**.

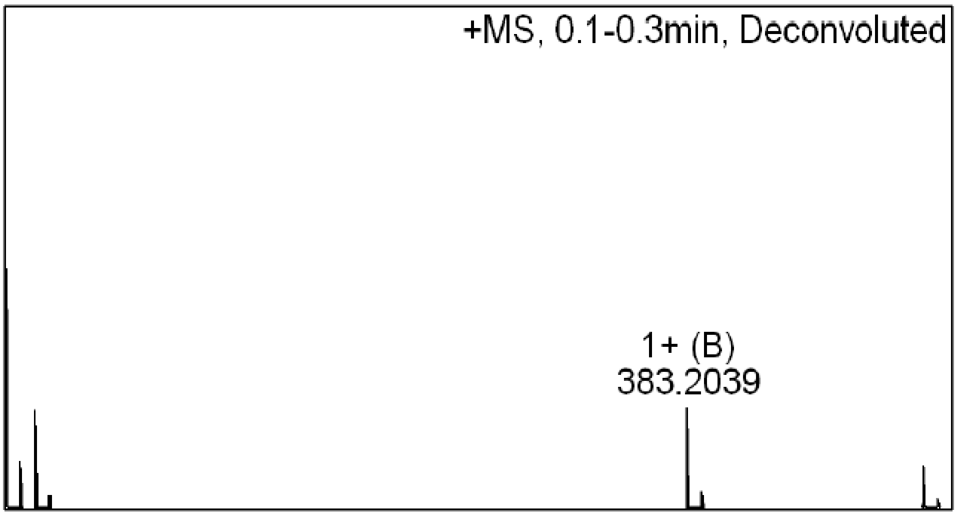

#	m/z	Res.	S/N	I	I %
1	337.1056			4991	100.0
2	339.1040			1938	38.8
3	383.2039			2001	40.1
4	399.1759			866	17.4

Figure S20. Copy of HRMS ESI analysis of the derivatives **7**.

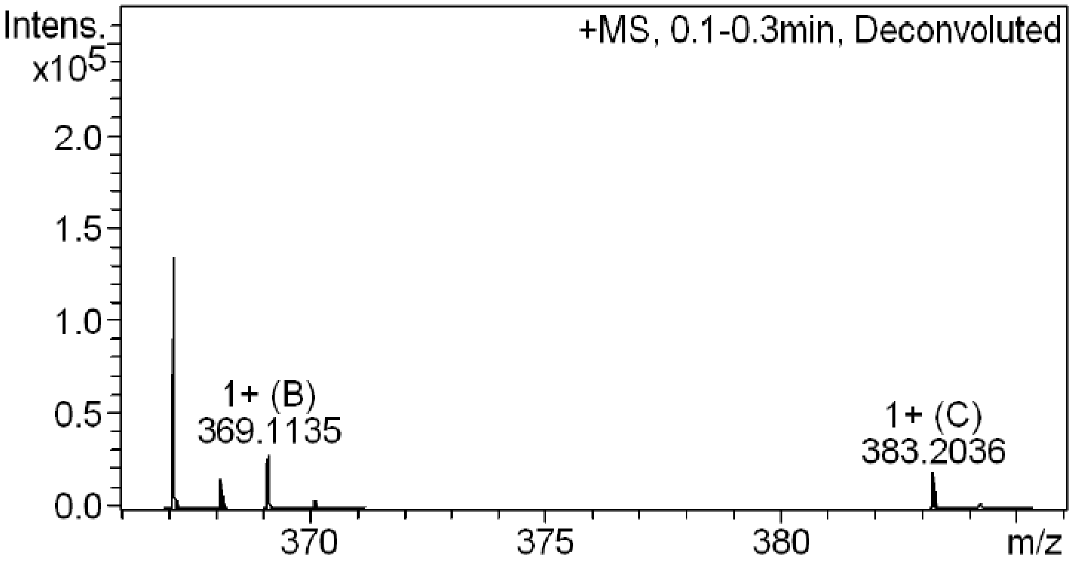

#	m/z	Res.	S/N	I	I %
1	367.1166			136969	100.0
2	369.1135			29731	21.7
3	383.2036			19119	14.0

Copies of HPLC Analysis

Figure S21. Copy HPLC analysis of the derivative **4**.

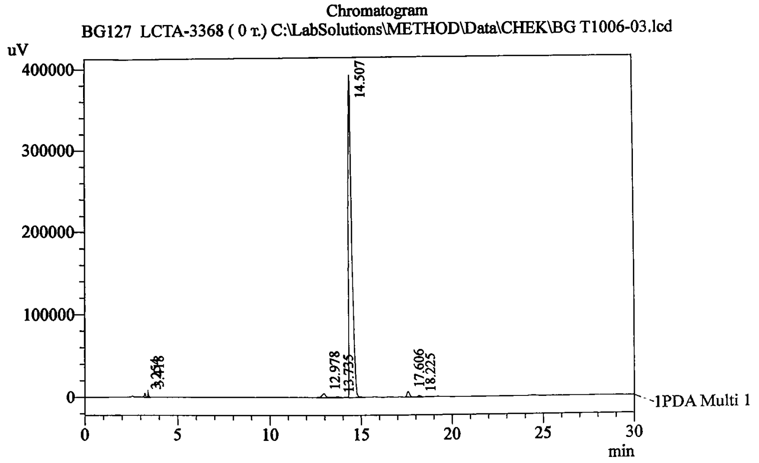

1 PDA Multi 1 / 274nm 4nm

PDA Ch1 274nm 4nm

Peak#	Ret. Time	Area	Height	Area %
1	3.254	23293	4606	0.453
2	3.418	24715	8545	0.480
3	12.978	63092	4781	1.226
4	13.735	11958	1018	0.232
5	14.507	4937973	390981	95.948
6	17.606	68747	6733	1.336
7	18.225	16713	1648	0.325
Total		5146490	418312	100.000

Method Filename : FOS Cv.lcm 06.10.2023 14:12:28

Time	Unit	Command	Valu
0.01	Pumps	B.Conc	10
30.00	Pumps	B.Conc	50
33.00	Pumps	B.Conc	10
45.00	Controller	Stop	

Shimadzu LC-20 AD; System - FOS Colon- Kromasil-100-5mkm. C-18, 4,6x250 mm. N 62511
Elution: A - H3PO4 0,01M pH 2,6; B - MeCN, fl - 1.0 ml/min, loop 20 mkl

Figure S22. Copy HPLC analysis of the derivative **5**.

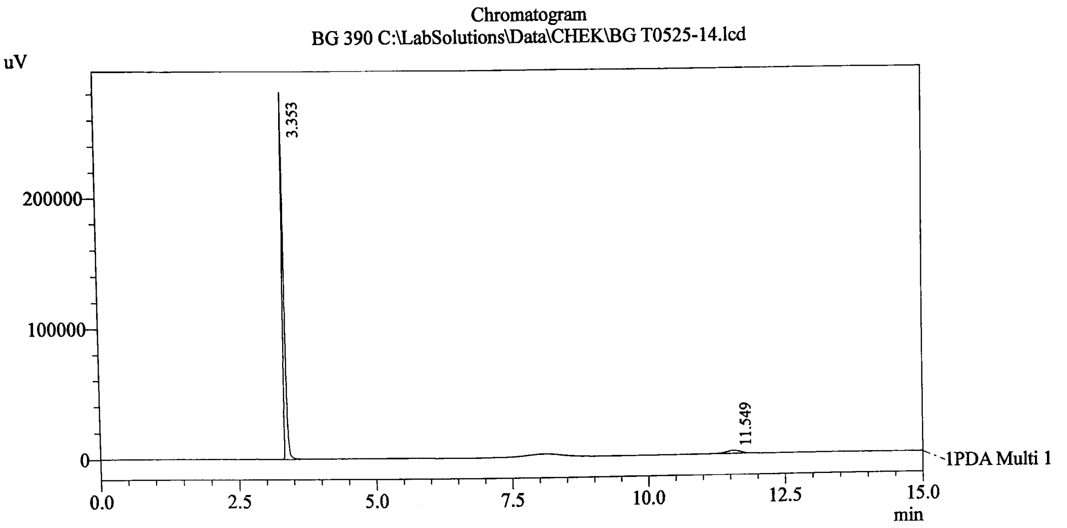

1 PDA Multi 1 / 377nm 4nm

PDA Ch1 377nm 4nm

Peak#	Ret. Time	Area	Height	Area %
1	3.353	678725	281537	95.011
2	11.549	35643	2099	4.989
Total		714367	283636	100.000

Method Filename : FRA02 vA.lcm 30.05.2023 13:58:19

Time	Unit	Command	Valu
0.01	Pumps	B.Conc	7
10.00	Pumps	B.Conc	7
30.00	Pumps	B.Conc	40
33.00	Pumps	B.Conc	7
45.00	Controller	Stop	

Shimadzu LC-20 AD; System - FRA 02 vA, Colon- Kromasil C-18, 4.6x250 mm. 5 mkm, N 59967
Elution: A - COOHNH4 0.2% pH 4.5; B - MeCN, fl - 1 ml/min, loop 20 mkl

Figure S23. Copy HPLC analysis of the derivative **6**.

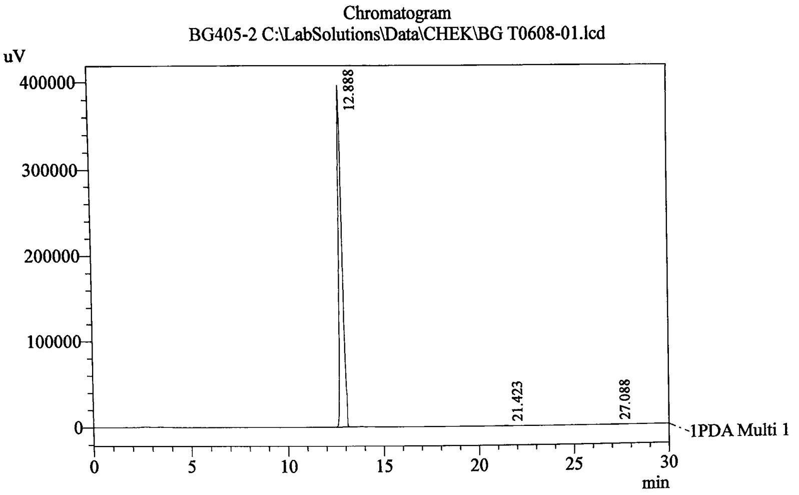

1 PDA Multi 1 / 375nm 4nm

PDA Ch1 375nm 4nm

Peak#	Ret. Time	Area	Height	Area %
1	12.888	7646484	395884	99.746
2	21.423	10493	639	0.137
3	27.088	8982	528	0.117
Total		7665960	397051	100.000

Method Filename : FOS Av.lcm 08.06.2023 12:02:02

Time	Unit	Command	Valu
0.01	Pumps	B.Conc	30
30.00	Pumps	B.Conc	50
35.00	Pumps	B.Conc	30
45.00	Controller	Stop	

Shimadzu LC-20 AD; System - FOS Colon- Kromasil-100-5mkm. C-18, 4,6x250 mm. N 62511
Elution: A - H3PO4 0,01M pH 2,6; B - MeCN, fl - 1.0 ml/min, loop 20 mkl

Figure S24. Copy HPLC analysis of the derivative **7**.

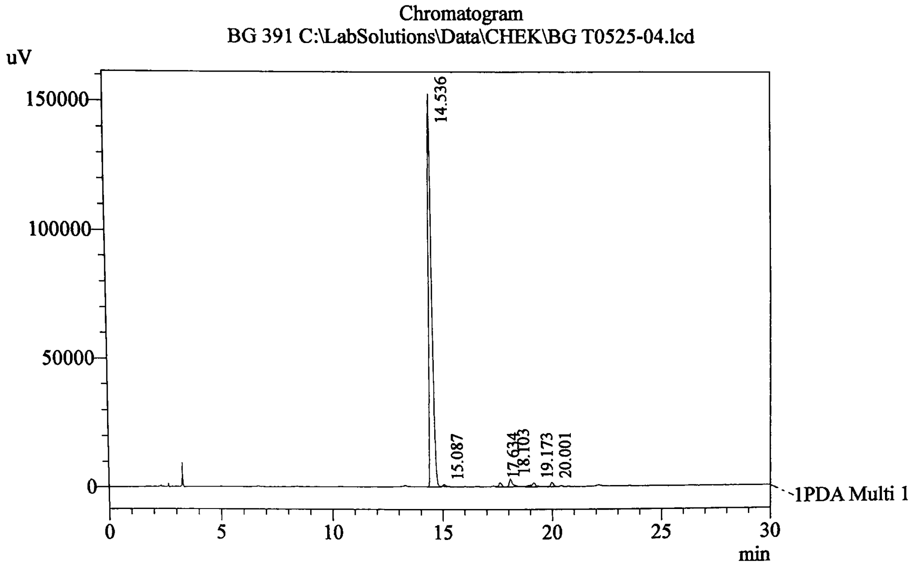

1 PDA Multi 1 / 370nm 4nm

PDA Ch1 370nm 4nm

Peak#	Ret. Time	Area	Height	Area %
1	14.536	1563763	152604	94.708
2	15.087	6165	769	0.373
3	17.634	14574	1585	0.883
4	18.103	30405	2944	1.841
5	19.173	20295	1489	1.229
6	20.001	15942	1686	0.966
Total		1651144	161077	100.000

Method Filename : FOS Av.lcm 26.05.2023 12:25:18

Time	Unit	Command	Valu
0.01	Pumps	B.Conc	10
30.00	Pumps	B.Conc	50
35.00	Pumps	B.Conc	10
45.00	Controller	Stop	

Shimadzu LC-20 AD; System - FOS Colon- Kromasil-100-5mkm. C-18, 4,6x250 mm. N 62511
Elution: A - H3PO4 0,01M pH 2,6; B - MeCN, fl - 1.0 ml/min, loop 20 mkl

Copies of IR Spectra

Figure S25. Copy of IR spectrum of the derivative **4**.

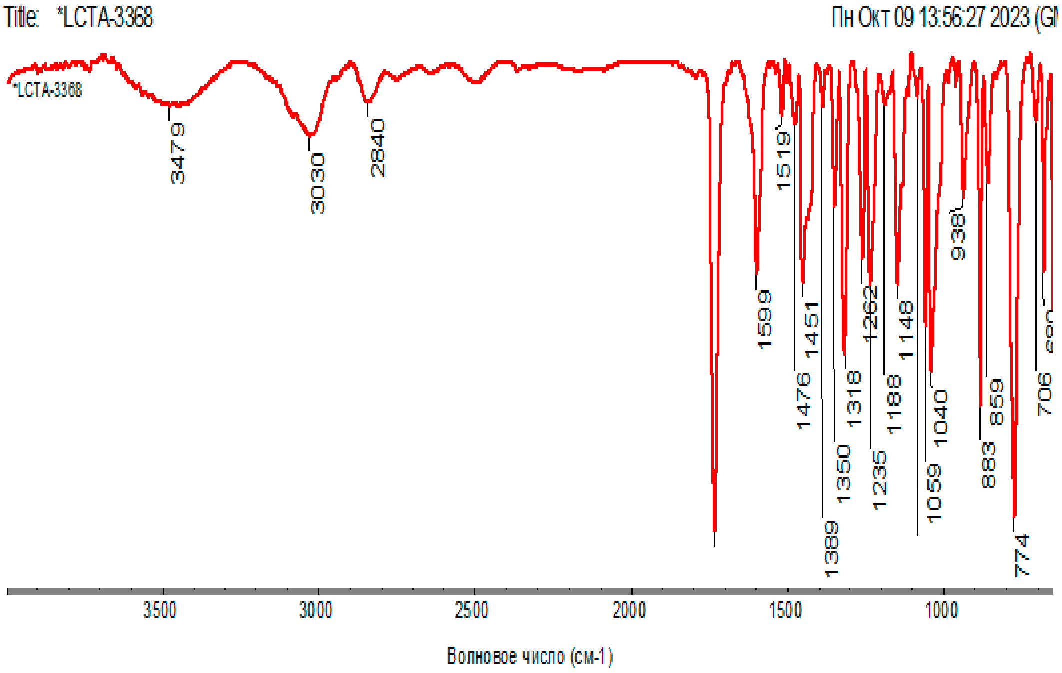

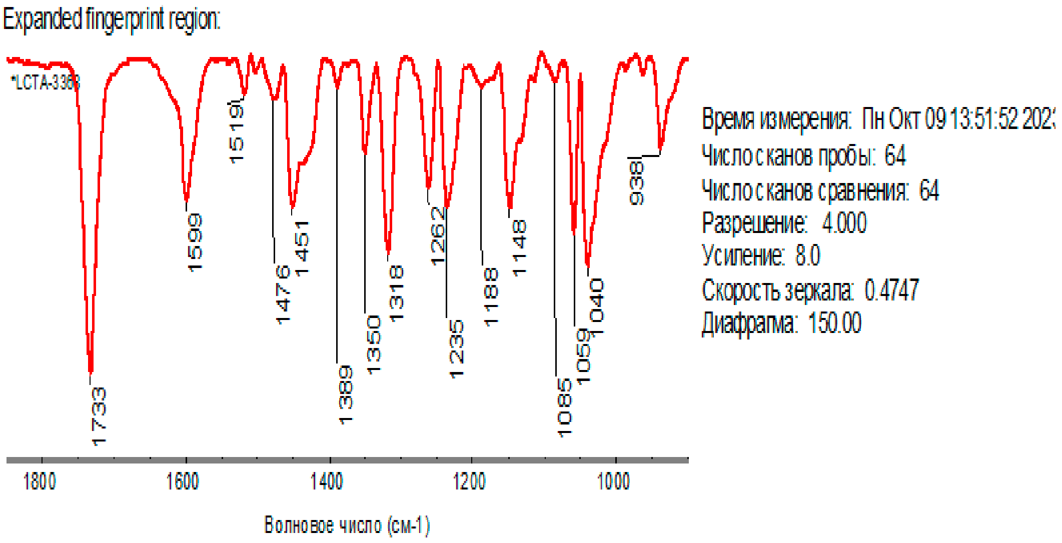

Figure S26. Copy of IR spectrum of the derivative **5**.

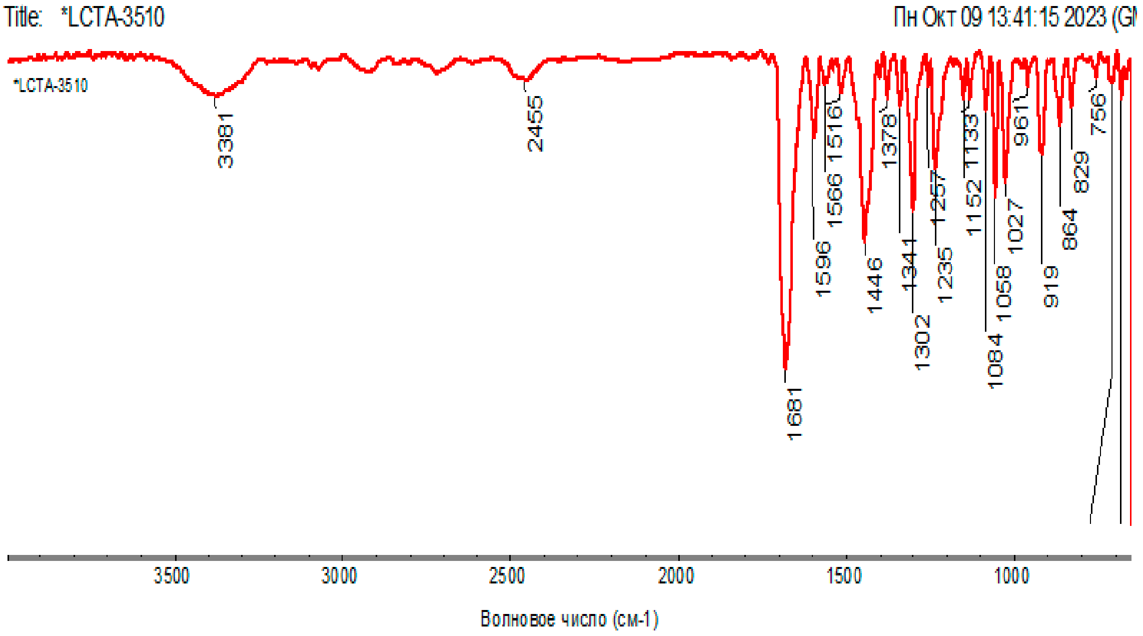

Expanded fingerprint region:

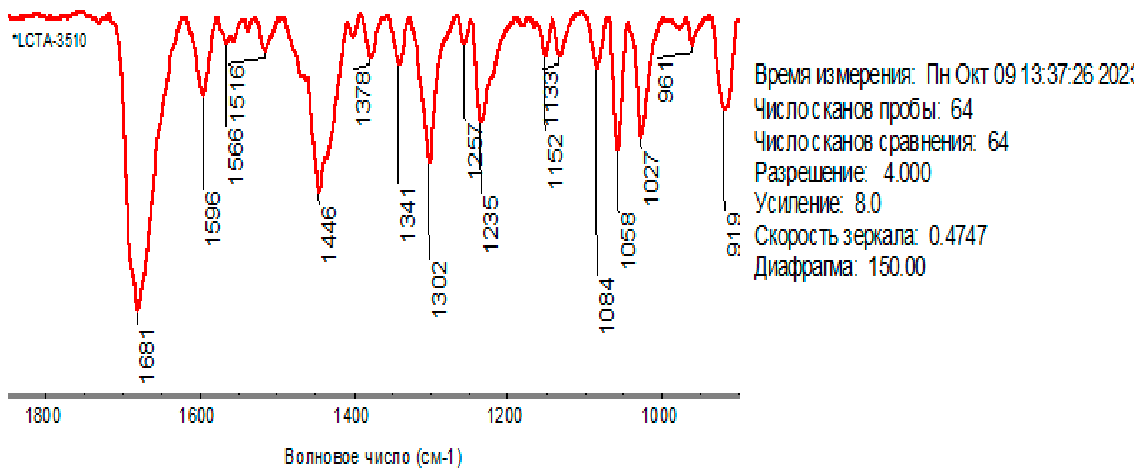

Figure S27. Copy of IR spectrum of the derivative **6**.

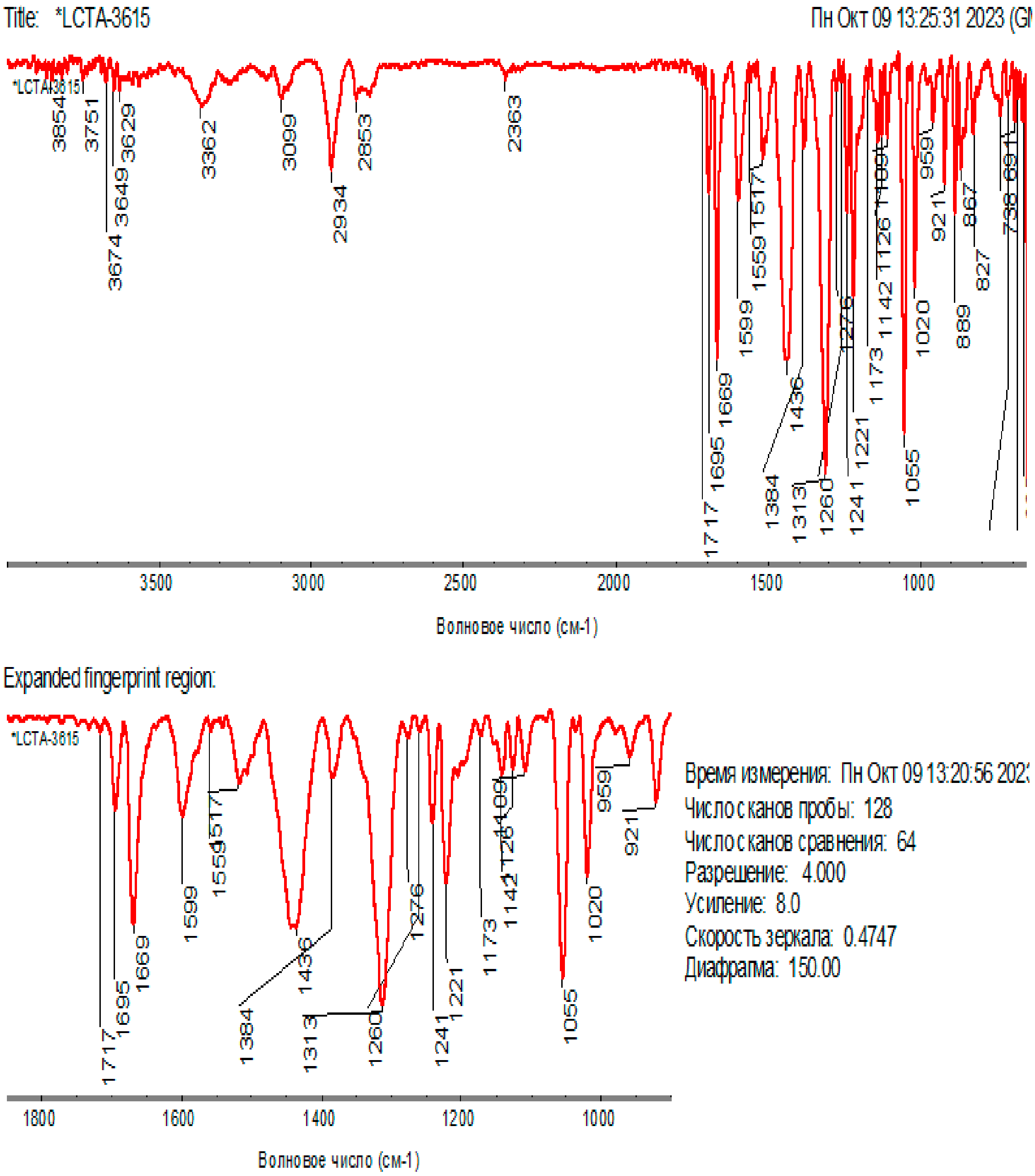

Figure S28. Copy of IR spectrum of the derivative **7**.

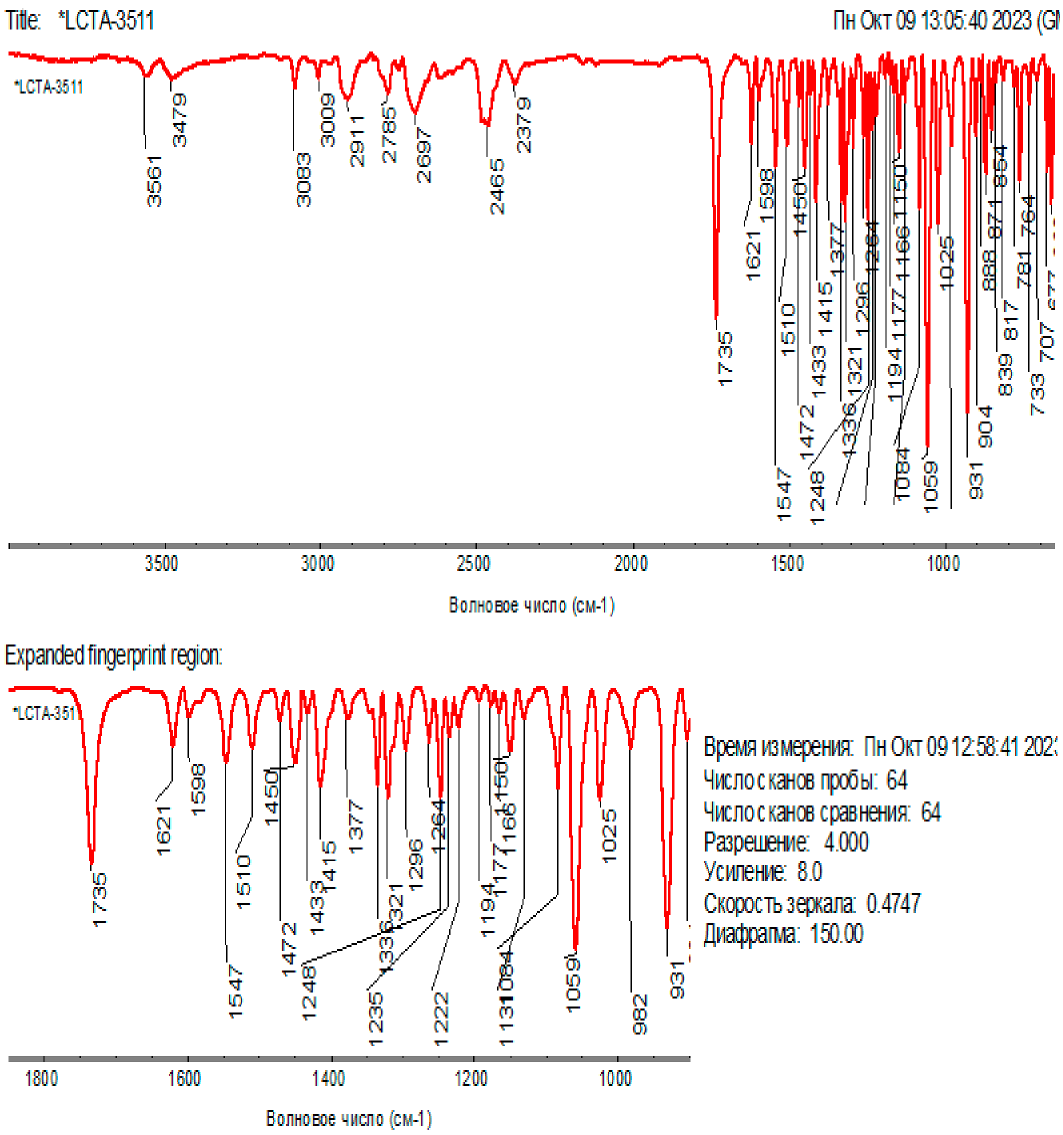

Copies of 2D NMR Spectra of 4 and 7.

Figure S29. ^{1}H-^{13}C HSQC spectrum for compound **4**.

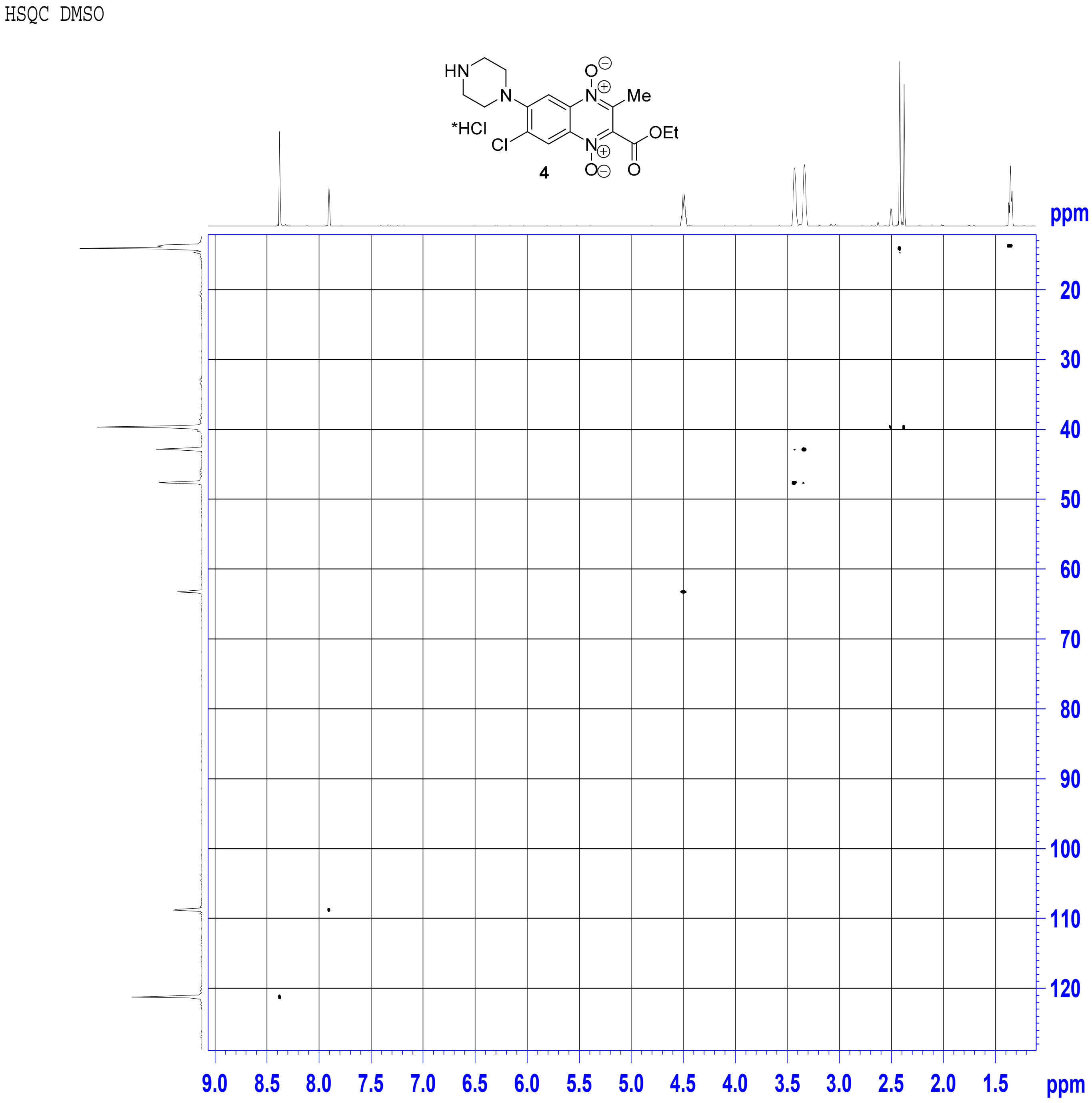

Figure S30. HMBC spectrum for compound **4**.

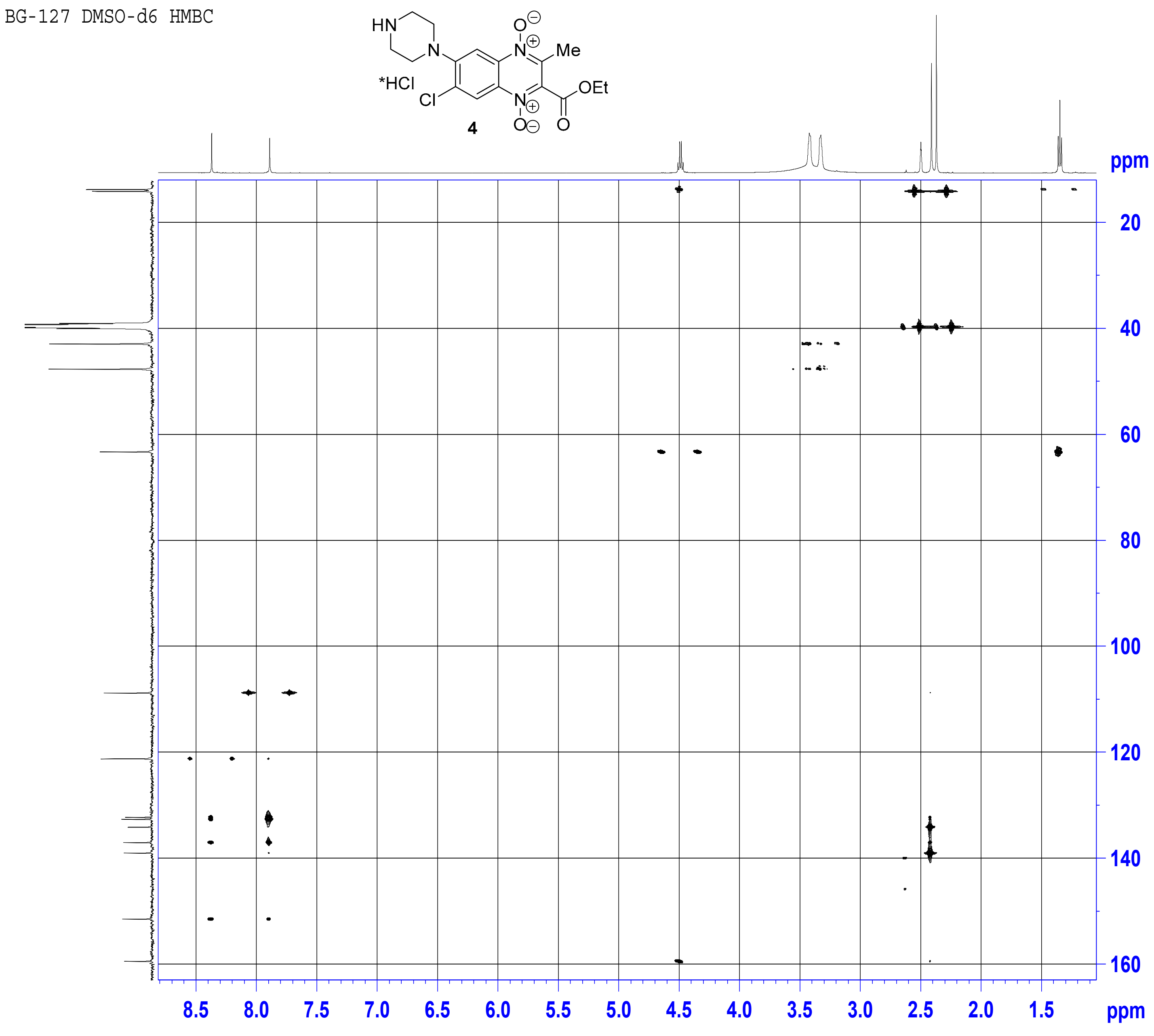

Figure S31. HMBC spectrum (fragment) for compound **4**.

BG-127 DMSO-d6 HMBC

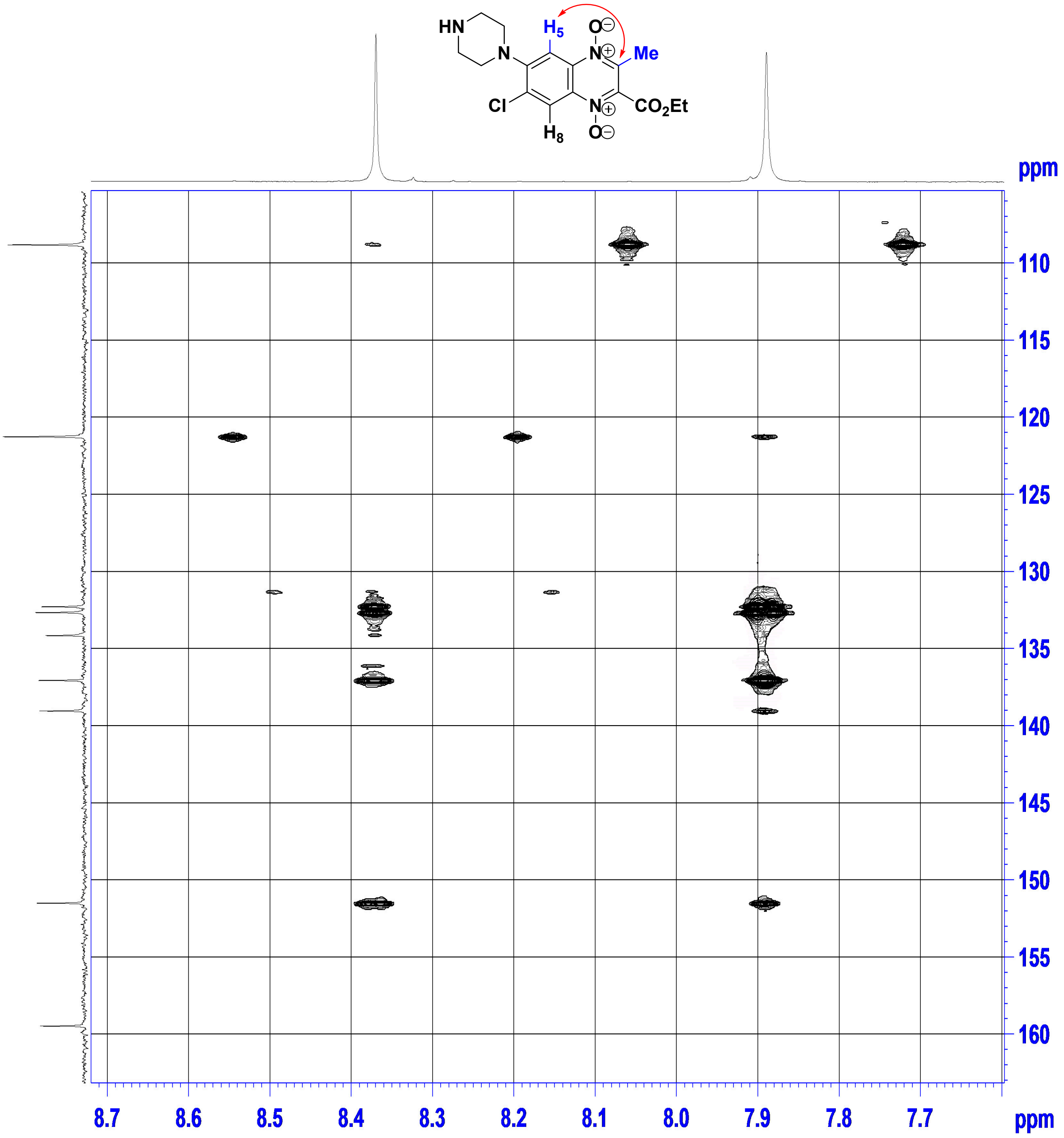

Figure S32. ^{1}H 2D NOESY spectrum of compound **4** in DMSO-d_6 at 500 MHz.

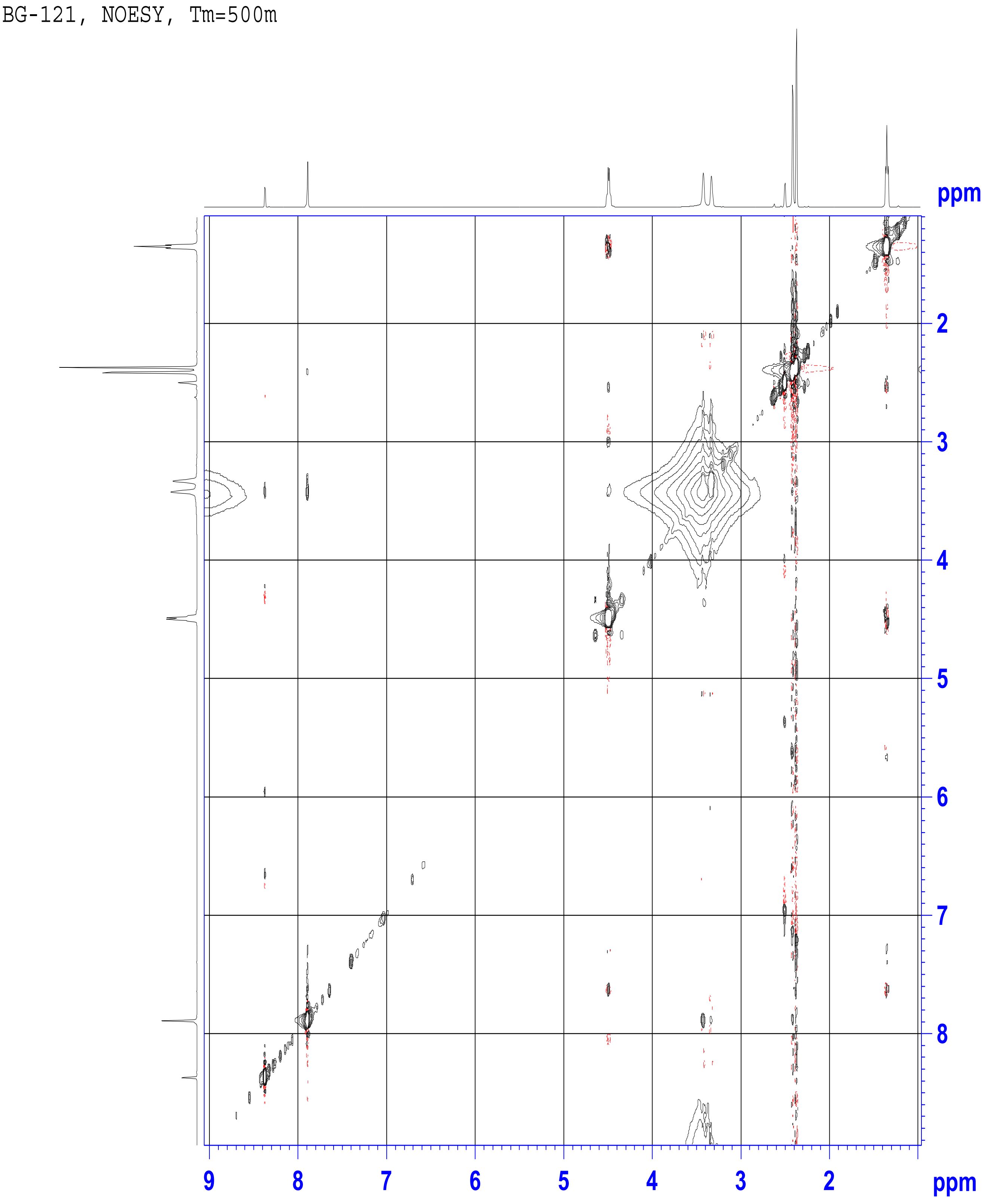

Figure S33. Selective NOE spectrum at CH_3 at 2.402 ppm for compound **4**.

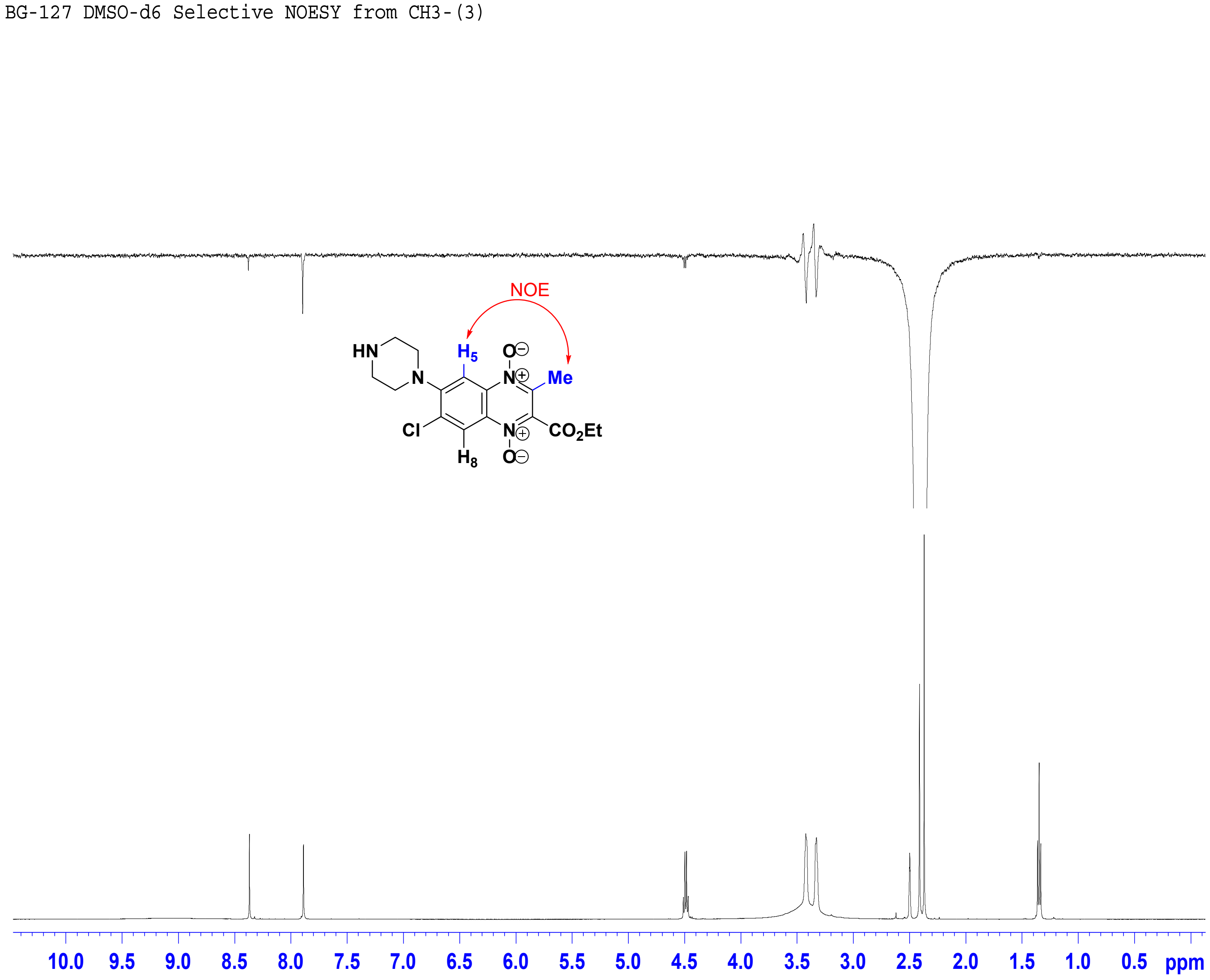

Figure S34. ^1H-^{13}C HSQC spectrum for compound **7**.

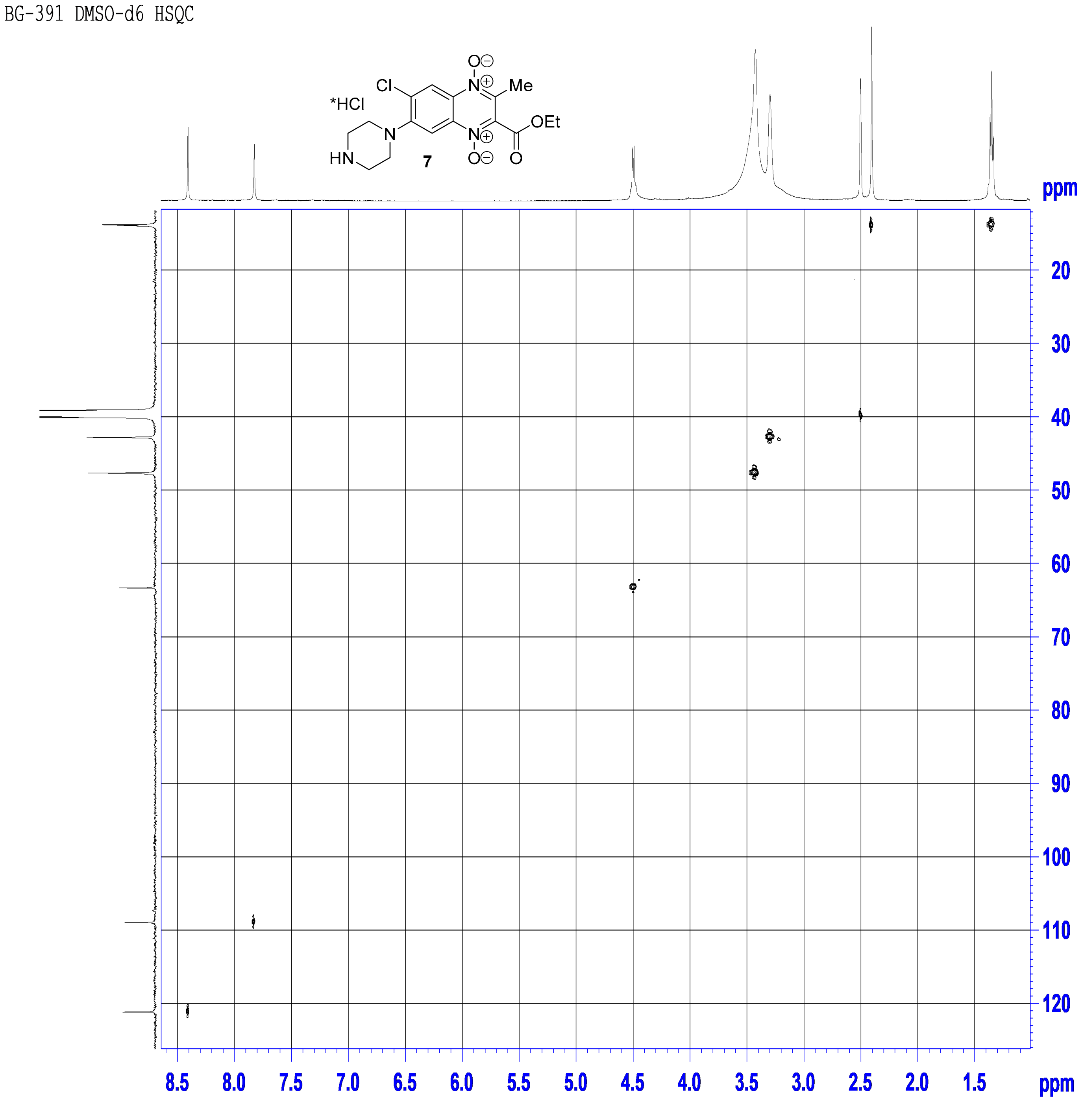

Figure S35. HMBC spectrum for compound **7**.

BG-391 DMSO-d6 HMBC

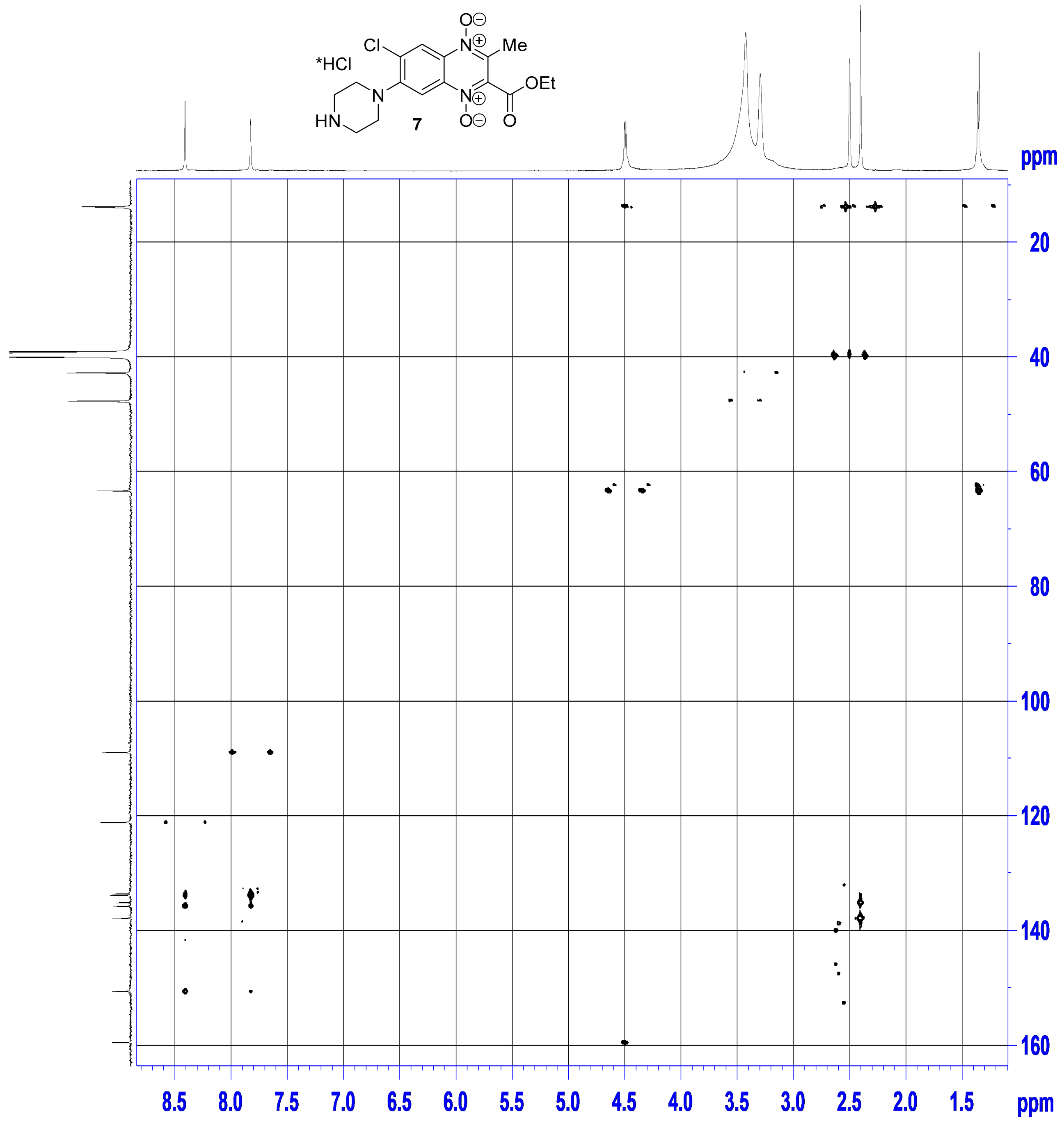

Figure S36. HMBC spectrum (fragment) for compound **7**.

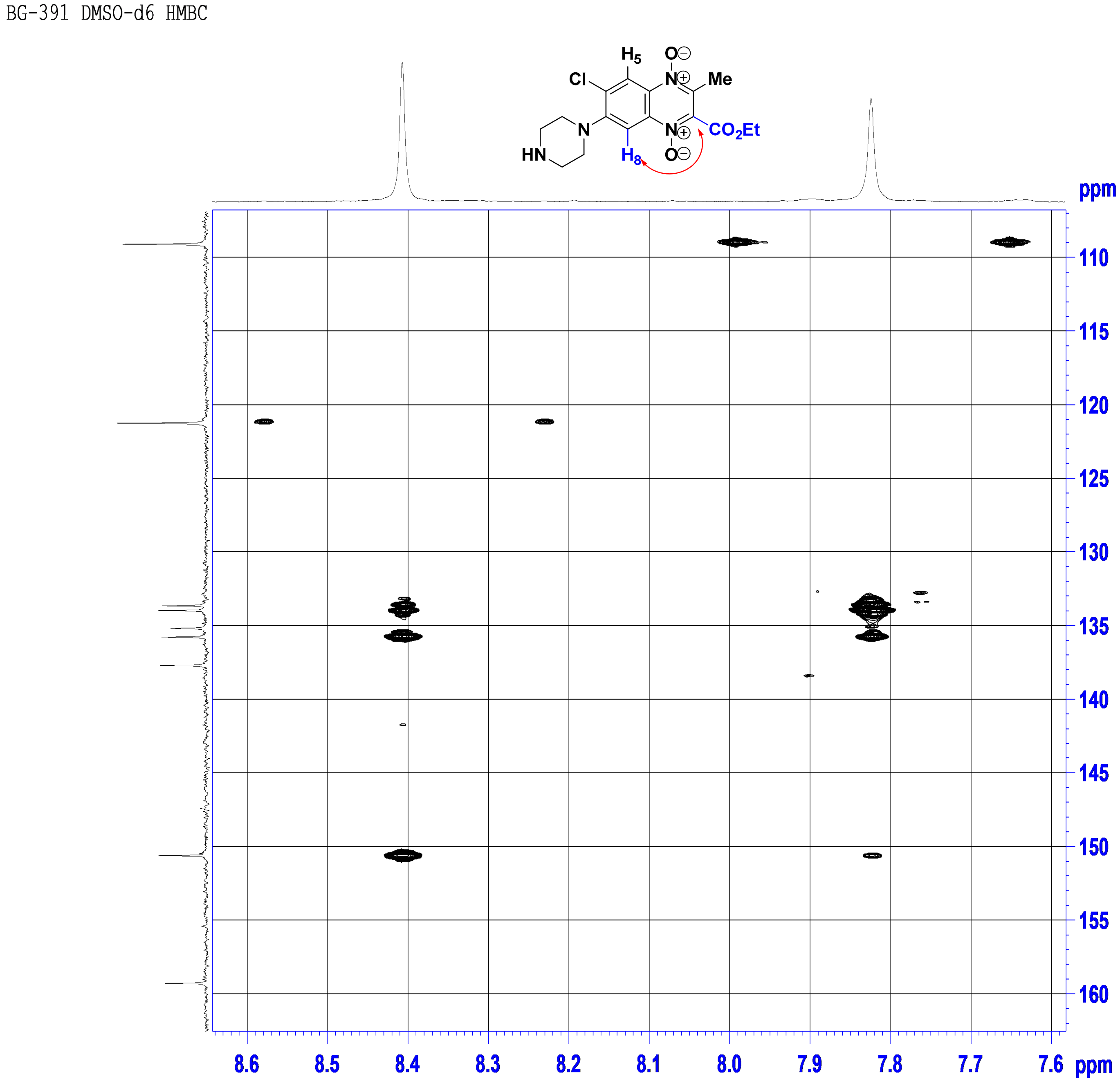

Figure S37. ^{1}H 2D NOESY spectrum of compound **7** in DMSO-*d₆* at 500 MHz.

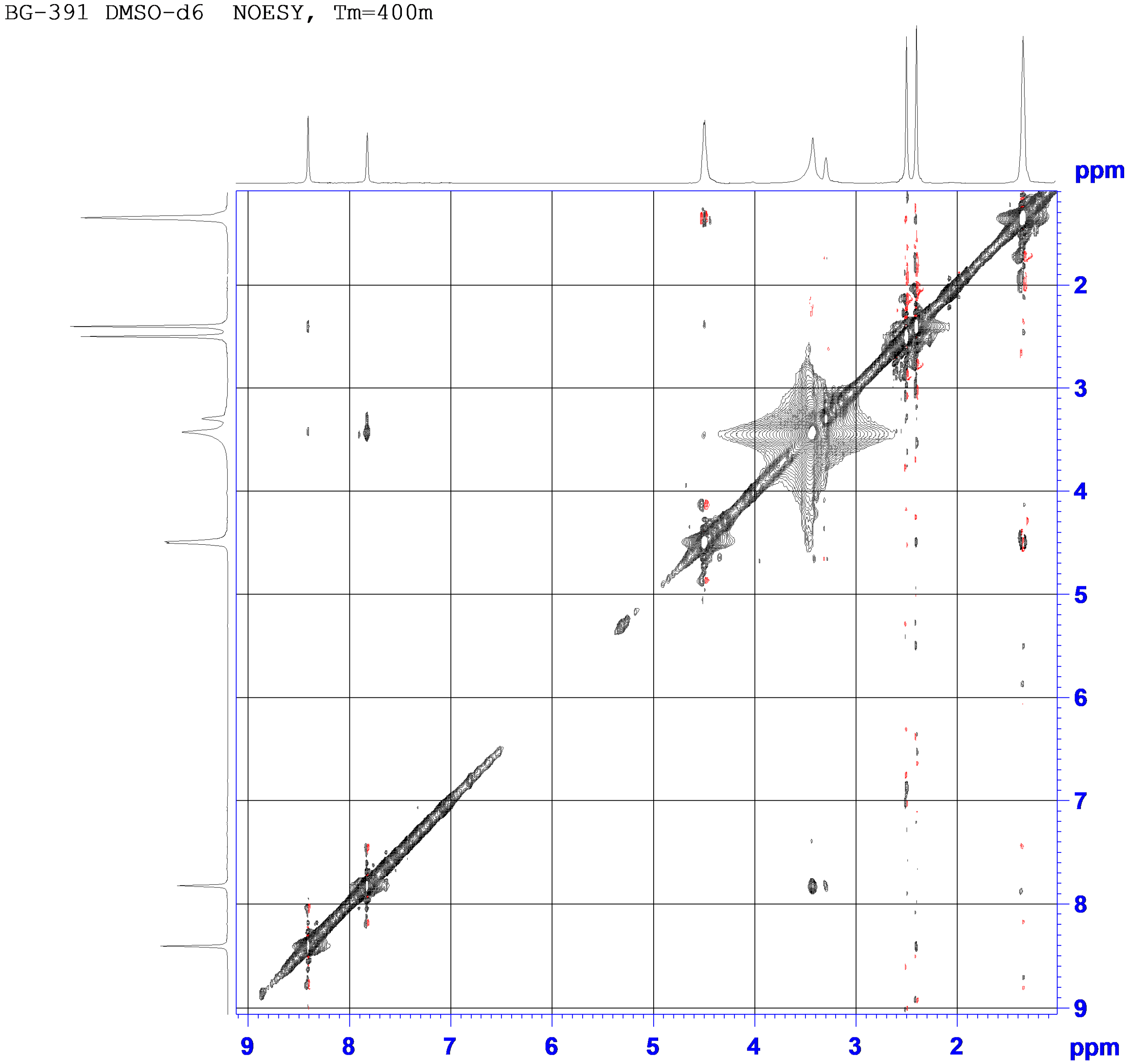

Figure S38. Selective NOE spectrum at CH_3 at 2.402 ppm for compound **7**.

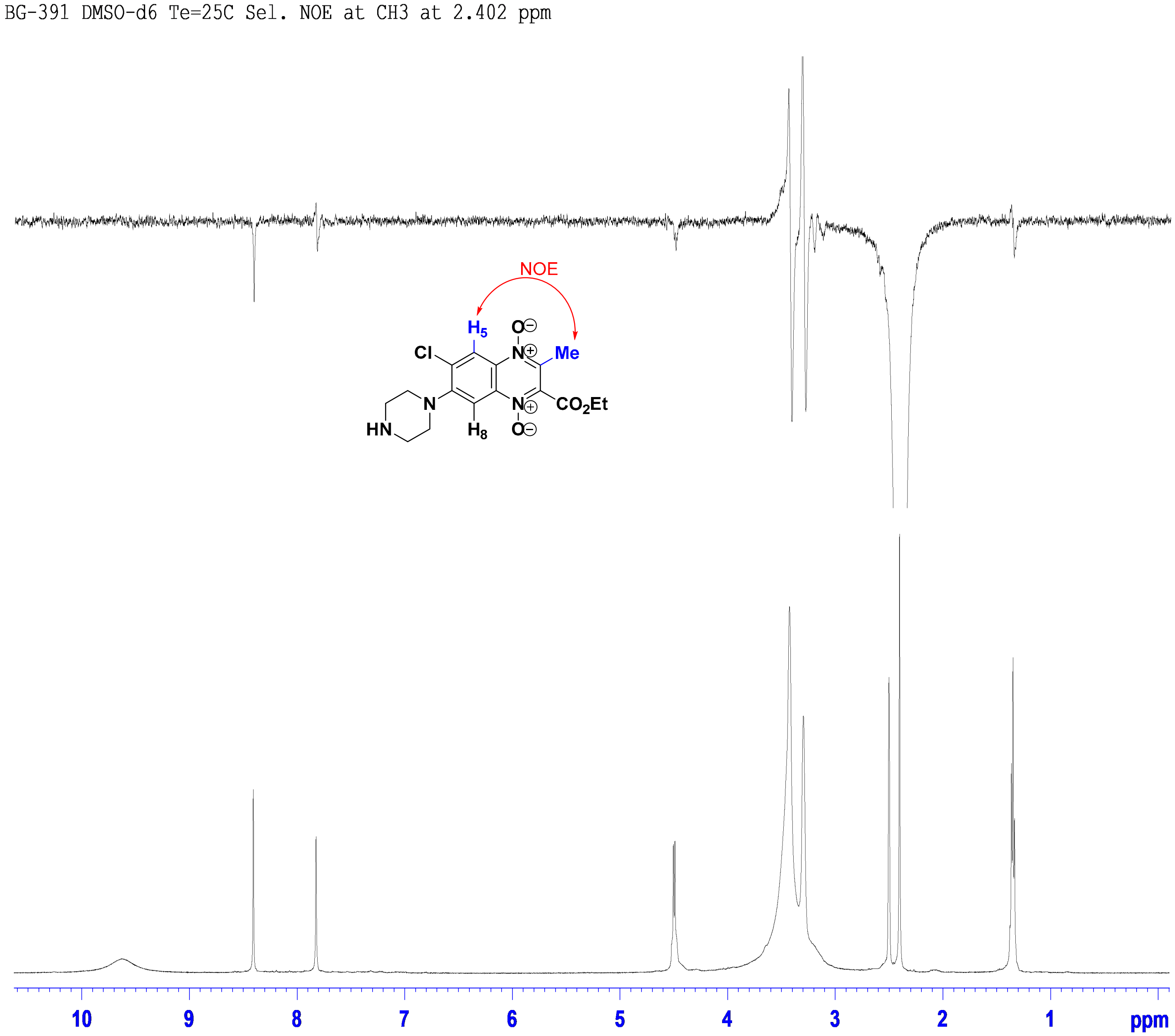

Table S1. ^{13}C chemical shifts (δ_C, ppm) and characteristic increments (I_C) for the ^{13}C chemical shift differences (relative to 2-ethoxycarbonyl-3-methylquinoxaline 1,4-dioxide) for the piperazine group for regioisomers **4** and **7**.

**Position**	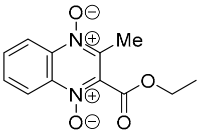	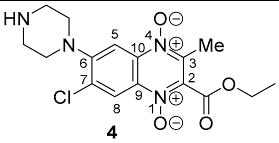 **4**		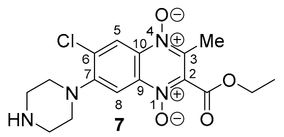 **7**	
	δ_C	δ_C	I^C	δ_C	I^C
C-2	134.8	134.1	−0.7	135.2	+0.4
C-3	138.3	139.0	+0.7	137.8	−0.5
C-9	137.4	132.3	−5.1	135.8	−1.6
C-10	136.2	137.0	+0.8	133.7	−2.5

Table S2. Stability of aqueous solutions of derivatives **4-7** after 7 days storage at 37 °C.

**Compound**	**HPLC analysis (purity, %)**	
	0 day	7 day
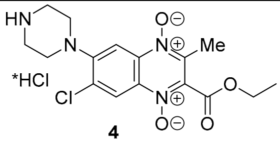 **4**	95.9	94.7
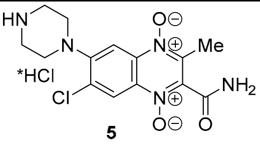 **5**	95.0	24.9
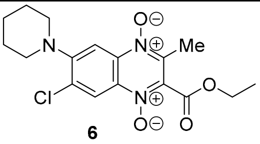 **6**	99.7	97.6
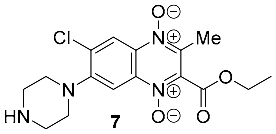 **7**	94.7	84.9

```
>MSMEG_4883
ATGAGCATATCGCTGCTGCTCGAGATGGCATCGTCGGGAGATCCCGACCGCACCGCGGTGGTTTCCGACG
ACACCCGGCTCACCGCAGGCGAGTTGAGCACATTGGCCGACGGGGCAGCAGGCGTCATCGCCGGGTCAGG
CGCGGCACACGTCGCCTACGTGGGTACCGGTGGCGCGCTGCTGCCGCTGCTGCTGTTCGCCTCGGCACGC
GCGGCGATCCCCTTCACCCCCCTGAACTACCGTCTCAGCGCCGAAGGTCTGCGCGAGCTGGTCGACCGGT
TGCCCACACCGCTGGTGATCGCCGACGGCGAGTACGCCGGCATGCTCGCCGGAGCGGGCAGGCCGGTGCT
CACCTCCGAGGAGTTCCTGTCCCAGGCCCGCACGGCCGATCCGGCCGCAGAGTTCGCCGACCCCGACGCG
GTCGCGGTGGTGCTGTTCACCTCGGGCACCACATCGCGCCCCAAGGCCGTCGAACTCACCCACAACAACC
TCACGAGCTACATCACCGGGACCGTCGAATTCGGTTCGGCCGCACCGGAGGACGCCGCGCTGATCTGTGT
GCCGCCGTACCACATCGCGGGTGTCAGCGCGGCGATGTCCAACCTGTACGCCGGCCGGAAGATGGTGTAC
CTGCGCAACTTCGACGCACACCGCTGGGTCGAGTTGGTGCGCACCGAGGGCGTCACGTCTGCCACCGTGG
TGCCGACCATGCTCGACCGTATCGTCACCGCACTTGAGACCACGCGCGCCGAGCTGCCGACCCTGCGCAA
CCTCGCCTACGGCGGATCCAAGGTCGCGCTGCCCCTGGTCCGCAAGGCGCTGGAGCTGATGCCGAACGTC
GGATTCGTCAACGCCTACGGTCTCACCGAAACCAGCTCCACCATCGCGGTCCTCGGACCCGACGACCACC
GGGCCGCGCTGGCCTCCGACGACCCCGGTGTGACCCGCCGCCTCGGGTCGGTGGGACAGGTGGTGCCCGG
GATCGAGGTGCAGATCCGCGGCGAGGACGGCACCGTGCTGGGGCCCGGCGAGACCGGTGAGCTGTTCGTG
CGCGGCGAGCAGGTGTCCGGCCGGTACACCGAGATCGGGTCGGTGCTCGACGAGGACGGCTGGTTCCCCA
CCAAAGACGTTGCGATGCTTGACCAGGACGGCTATCTGTTCATCGGCGGCCGGTCGGACGACACCATCAT
CCGCGGCGGCGAGAACATCGCTCCCGCCGAGATCGAGGACGTTCTCGTGGAACATCCCGACGTGCGCGAC
GTCGCGGTGGTCGGCCCCGAGGACCCCCAGTGGGGCCAGATCATCGTCGCGGTCGTGGTGCCCGCCGACG
GCGCCGAACCCGACGCCGACGTACTCCGCGAGCACGTCCGCAAACACCTGCGCGGATCCCGCACCCCCGA
CCGCGTGGTCTTCCGCGCCGAACTGCCCACCAACGCCACCGGCAAGGTGCTGCGCCGTCAACTCGTCGAC
GAACTCCAGCCCATCTCGTAG
```

Figure S39. Nucleotide sequence of MSMEG_4883. The 9 bp repeats are underlined, while the deletion region is highlighted as bold.

Table S3. Unique SNPs in quinoxaline-resistant *M. smegmatis* mutants.

M. smegmatis qdr1					
Locus tag	Protein ID	annotation	Codon	SNP	Amino acid substitution
MSMEG_0210	YP_884625.1	LprO protein	codon=45	CTG>CTT	L>L
MSMEG_0210	YP_884625.1	LprO protein	codon=45	CTG>CCG	L>P
MSMEG_0229	YP_884644.1	dihydroxy-acid dehydratase	codon=38	ATG>ATT	M>I
MSMEG_0232	YP_884647.1	sugar transporter family protein	codon=349	GGC>GAC	G>D
MSMEG_0234	YP_884649.1	metallopeptidase	codon=158	CTG>CCG	L>P
MSMEG_0318	YP_884733.1	AMP-dependent synthetase/ligase	codon=23	CCT>CCA	P>P
MSMEG_0613	YP_885024.1	hypothetical protein	codon=148	ATG>ACG	M>T
MSMEG_0705	YP_885112.1	permease of the major facilitator superfamily protein	codon=119	CTG>CGG	L>R
MSMEG_0706	YP_885113.1	4-carboxy-4-hydroxy-2-oxoadipate aldolase	codon=38	GGC>AGC	G>S
MSMEG_1240	YP_885631.1	hypothetical protein	codon=253	CAT>CGT	H>R
MSMEG_1328	YP_885718.1	exodeoxyribonuclease V subunit gamma	codon=236	ACT>GCT	T>A
MSMEG_1380	YP_885766.1	transcriptional regulator	-	-	-
MSMEG_1684	YP_886060.1	hypothetical protein	codon=207	GAG>CAG	E>Q
MSMEG_1775	YP_886147.1	cytochrome P450 monooxygenase	Intergenic mutation		
MSMEG_1930	YP_886296.1	DEAD/DEAH box helicase	Intergenic mutation		
MSMEG_2257	YP_886609.1	cytochrome P450-terp	codon=394	GCC>GGC	A>G
MSMEG_4378	YP_888653.1	two-component system response regulator	codon=109	GTG>GGG	V>G
MSMEG_4648	YP_888911.1	DNA-binding protein	codon=49	CAG>CCG	Q>P
MSMEG_4702	YP_888963.1	ABC transporter permease	codon=73	GCG>GGG	A>G
MSMEG_4712	YP_888972.1	pyruvate dehydrogenase E1 component subunit alpha	codon=160	CAG>CGG	Q>R
MSMEG_4846	YP_889102.1	enoyl-CoA hydratase	Intergenic mutation		
MSMEG_4861	YP_889117.1	cyclase	Intergenic mutation		
MSMEG_5122	YP_889369.1	ferredoxin	Intergenic		

			mutation		
MSMEG_5122	YP_889369.1	ferredoxin	Intergenic mutation		
MSMEG_5348	YP_889592.1	medium-chain fatty acid-CoA ligase	codon=254	CCC>CGC	P>R
MSMEG_5351	YP_889595.1	hypothetical protein	codon=43	GCG>GGG	A>G
MSMEG_5437	YP_889676.1	serine/threonine protein kinase	codon=272	CAT>TAT	H>Y
MSMEG_5600	YP_889834.1	hypothetical protein	Intergenic mutation		
MSMEG_5757		tRNA-Glu	codon=22	GTT>GCT	V>A
MSMEG_6041	YP_890265.1	acyl-CoA dehydrogenase	codon=281	ATC>GTC	I>V
MSMEG_6232	YP_890451.1	catalase KatA	codon=115	GGG>CGG	G>R
MSMEG_6241	YP_890460.1	ATPase AAA	codon=194	GTG>GCG	V>A
MSMEG_6317	YP_890535.1	lipolytic protein G-D-S-L	codon=132	CCC>CCA	P>P
MSMEG_6628	YP_890837.1	TetR family transcriptional regulator	Intergenic mutation		
MSMEG_6858	YP_891062.1	epoxide hydrolase 1	codon=163	TCG>TTG	S>L

M. smegmatis qdr2

Locus tag	Protein ID	annotation	Codon	SNP	Amino acid substitution
MSMEG_0001	YP_884424.1	DNA polymerase III subunit beta	codon=152	GCG>GGG	A>G
MSMEG_0312	YP_884726.1	2-dehydro-3-deoxy-phosphogluconate aldolase	codon=99	ATG>ACG	M>T
MSMEG_0576	YP_884987.1	MmpL4 protein	codon=377	GTG>GTT	V>V
MSMEG_0634	YP_885043.1	PAP2 superfamily protein	codon=37	GAC>GGC	D>G
MSMEG_0660	YP_885069.1	extracellular solute-binding protein	codon=158	TTG>TTA	L>L
MSMEG_0719	YP_885126.1	flavohemoprotein	codon=175	CAG>CGG	Q>R
MSMEG_1080	YP_885476.1	large subunit of N,N-dimethylformamidase	codon=490	ACA>ACG	T>T
MSMEG_1111	YP_885505.1	hypothetical protein	codon=6	GCC>GGC	A>G
MSMEG_1119	YP_885513.1	glutamine amidotransferase	codon=114	CGA>AGA	R>R
MSMEG_1211	YP_885603.1	Fatty acid desaturase	Intergenic mutation		
MSMEG_1380	YP_885766.1	transcriptional regulator	-	-	-
MSMEG_1476	YP_885858.1	signal peptide peptidase SppA, 67K type	codon=232	GAG>GGG	E>G

MSMEG_1476	YP_885858.1	signal peptide peptidase SppA, 67K type	codon=420	AAA>AAC	K>N
MSMEG_1641	YP_886018.1	hypothetical protein	codon=181	GCC>GCT	A>A
MSMEG_1644	YP_886021.1	hypothetical protein	codon=247	GTG>ATG	V>M
MSMEG_2077	YP_886438.1	acyl-CoA dehydrogenase	codon=145	GTC>CTC	V>L
MSMEG_2512	YP_886850.1	lactate 2-monooxygenase	codon=241	ACC>ACG	T>T
MSMEG_2968	YP_887287.1	hypothetical protein	codon=144	AAC>AAT	N>N
MSMEG_3159	YP_887474.1	methylmalonyl-CoA mutase	codon=28	CCC>CCG	P>P
MSMEG_3975	YP_888263.1	regulatory protein	codon=396	GAG>GAC	E>D
MSMEG_4044	YP_888330.1	GAF domain-containing protein	codon=467	CAG>CGG	Q>R
MSMEG_4723	YP_888983.1	hypothetical protein	codon=365	GTG>GCG	V>A
MSMEG_4853	YP_889109.1	peptidase, M24 family protein	codon=10	CGG>CGC	R>R
MSMEG_4872	YP_889128.1	short chain dehydrogenase	Intergenic mutation		
MSMEG_5122	YP_889369.1	ferredoxin	codon=5	ATT>ACT	I>T
MSMEG_5122	YP_889369.1	ferredoxin	codon=29	CAT>TAT	H>Y
MSMEG_5152	YP_889397.1	hypothetical protein	codon=29	CAT>TAT	H>Y
MSMEG_5423	YP_889663.1	transcription-repair coupling factor	codon=906	ATC>ATT	I>I
MSMEG_5619	YP_889853.1	acyl-CoA dehydrogenase	codon=166	GAC>GGC	D>G
MSMEG_5656	YP_889889.1	histidinol dehydrogenase	codon=422	GCC>GCT	A>A
MSMEG_6313	YP_890531.1	queuine tRNA-ribosyltransferase	codon=349	ACG>CCG	T>P
MSMEG_6341	YP_890558.1	6-phosphogluconate dehydrogenase	codon=190	GTG>GTC	V>V

M. smegmatis qdr3

Locus tag	Protein ID	annotation	Codon	SNP	Amino acid substitution
MSMEG_0017	YP_884437.1	ABC transporter permease	codon=535	AGT>CGT	S>R
MSMEG_1186	YP_885578.1	permease	codon=431	ATC>CTC	I>L
MSMEG_1325	YP_885716.1	exodeoxyribonuclease V subunit alpha	codon=419	GAA>AAA	E>K
MSMEG_1380	YP_885766.1	transcriptional regulator	codon=54	TAC>TCC	Y>S
MSMEG_1469	YP_885851.1	30S ribosomal protein S8	codon=58	AAG>ACG	K>T
MSMEG_1515	YP_885897.1	two-component sensor histidine kinase	codon=137	GGC>GAC	G>D
MSMEG_2115	YP_886471.1	hypothetical protein	codon=95	GAC>TAC	D>Y

MSMEG_2115	YP_886471.1	hypothetical protein	codon=95	GAC>GCC	D>A
MSMEG_2139	YP_886496.1	hypothetical protein	Intergenic mutation		
MSMEG_2350	YP_886693.1	hypothetical protein	codon=223	CTC>CCC	L>P
MSMEG_2580	YP_886917.1	4-hydroxy-3-methylbut-2-en-1-yl diphosphate synthase	codon=69	GTG>GTA	V>V
MSMEG_2907	YP_887226.1	oxidoreductase YeiQ	codon=443	GTC>CTC	V>L
MSMEG_2950	YP_887269.1	carbon monoxide dehydrogenase, medium chain	codon=5	CGG>GGG	R>G
MSMEG_3673	YP_887976.1	4-alpha-glucanotransferase	codon=272	GTG>GTA	V>V
MSMEG_3741	YP_888040.1	transcriptional regulator	codon=153	AAA>AAG	K>K
MSMEG_4108	YP_888390.1	NAD(P) transhydrogenase subunit beta	codon=370	CTG>CTC	L>L
MSMEG_4109	YP_888391.1	NAD(P) transhydrogenase subunit alpha	codon=408	GGA>GCA	G>A
MSMEG_4259	YP_888535.1	hypothetical protein	codon=280	CTG>CCG	L>P
MSMEG_4383	YP_888658.1	MmpL5 protein	codon=66	GTG>GGG	V>G
MSMEG_4384	YP_888659.1	penicillin binding protein transpeptidase domain-containing protein	codon=41	GTG>GTA	V>V
MSMEG_4477	YP_888750.1	hydrolase, alpha/beta hydrolase fold family protein	codon=236	CTC>CTG	L>L
MSMEG_4641	YP_888903.1	salicylate hydroxylase	codon=234	CGC>CCC	R>P
MSMEG_4646	YP_888909.1	pyruvate synthase	codon=652	TGA>TCA	*>S
MSMEG_4917	YP_889173.1	hypothetical protein	codon=73	GCC>TCC	A>S
MSMEG_5585	YP_889821.1	hypothetical protein	codon=39	GCC>GGC	A>G
MSMEG_6749	YP_890957.1	hypothetical protein	codon=11	AAG>AGG	K>R
MSMEG_6934	YP_891128.1	thioredoxin	codon=90	GTG>GGG	V>G

M. smegmatis qdr4

Locus tag	Protein ID	annotation	Codon	SNP	Amino acid substitution
MSMEG_0166	YP_884582.1	GntR family transcriptional regulator	Intergenic mutation		
MSMEG_0204	YP_884619.1	hypothetical protein	codon=610	GCG>GTG	A>V
MSMEG_0217	YP_884632.1	alcohol dehydrogenase	Intergenic mutation		

MSMEG_0635	YP_885045.1	hypothetical protein	codon=93	ACC>AAC	T>N
MSMEG_1088	YP_885483.1	glutamyl-tRNA(Gln)/aspartyl-tRNA(Asn) amidotransferase subunit alpha	Intergenic mutation		
MSMEG_1915	YP_886281.1	anti-sigma factor, family protein	codon=55	TGC>TGG	C>W
MSMEG_2401	YP_886740.1	hypothetical protein	codon=170	CCG>GCG	P>A
MSMEG_2531	YP_886868.1	GntR family transcriptional regulator	Intergenic mutation		
MSMEG_3598	YP_887901.1	periplasmic sugar-binding proteins	codon=253	GAC>GTC	D>V
MSMEG_3878	YP_888169.1	precorrin-6Y C5,15-methyltransferase	codon=343	GCG>GCC	A>A
MSMEG_4147	YP_888427.1	major facilitator superfamily protein	codon=8	GTC>TTC	V>F
MSMEG_4290	YP_888567.1	glutamine synthetase	Intergenic mutation		
MSMEG_4646	YP_888909.1	pyruvate synthase	codon=95	AAC>CAC	N>H
MSMEG_5117	YP_889365.1	proline dehydrogenase	codon=184	GAG>CAG	E>Q
MSMEG_5311	YP_889557.1	hypothetical protein	codon=133	GCG>TCG	A>S
MSMEG_5798	YP_890026.1	phosphoribosylaminoimidazole synthetase	codon=336	TGG>CGG	W>R
MSMEG_5836	YP_890063.1	hypothetical protein	codon=141	CAC>TAC	H>Y
MSMEG_6636	YP_890845.1	[Mn] superoxide dismutase	Intergenic mutation		

M. smegmatis qdr5

Locus tag	Protein ID	annotation	Codon	SNP	Amino acid substitution
MSMEG_0070	YP_884488.1	hypothetical protein	Intergenic mutation		
MSMEG_0125	YP_884540.1	integral membrane protein	codon=190	TCG>CCG	S>P
MSMEG_0144	YP_884561.1	HNH endonuclease	codon=417	GAC>GTC	D>V
MSMEG_0354	YP_884767.1	hypothetical protein	codon=104	GCA>CCA	A>P
MSMEG_0516	YP_884928.1	sugar transporter	codon=176	ATC>ATG	I>M
MSMEG_0772	YP_885178.1	phytase	Intergenic mutation		
MSMEG_1380	YP_885766.1	transcriptional regulator	codon=108	GGA>TGA	G>*
MSMEG_1559	YP_885939.1	phosphoglucosamine mutase	codon=148	CGG>CCG	R>P
MSMEG_2148	YP_886504.1	HNH endonuclease domain-containing protein	Intergenic mutation		

MSMEG_2349	YP_886692.1	glycosyl hydrolase family protein	codon=85	ATG>GTG	M>V
MSMEG_2377	YP_886719.1	P49 protein	codon=178	GTT>TTT	V>F
MSMEG_3143	YP_887458.1	aconitate hydratase	codon=835	GGC>GGG	G>G
MSMEG_3183	YP_887498.1	threonine dehydratase	codon=2	ACC>GCC	T>A
MSMEG_3281	YP_887592.1	spermidine/putrescine ABC transporter ATP-binding protein	codon=184	GGC>GGA	G>G
MSMEG_3815	YP_888107.1	drug efflux membrane protein	codon=29	GCC>GGC	A>G
MSMEG_3839	YP_888130.1	DNA polymerase I	codon=242	GGC>TGC	G>C
MSMEG_3872	YP_888163.1	precorrin-8X methylmutase	codon=44	GGC>GGA	G>G
MSMEG_4516	YP_888788.1	2,3-dihydroxybenzoate-AMP ligase	codon=359	CTG>CTC	L>L
MSMEG_4646	YP_888909.1	pyruvate synthase	codon=274	CCG>CTG	P>L
MSMEG_4721	YP_888981.1	permease of the major facilitator superfamily protein	codon=114	GTG>GGG	V>G
MSMEG_4762	YP_889019.1	ABC transporter	codon=112	TCG>TTG	S>L
MSMEG_4793	YP_889049.1	virulence factor Mce family protein	codon=69	GTT>GTC	V>V
MSMEG_5187	YP_889433.1	tetracycline-resistance determinant TetV	codon=82	AAC>AAG	N>K
MSMEG_5253	YP_889499.1	hypothetical protein	codon=2	ACC>ATC	T>I
MSMEG_5450	YP_889689.1	redox-sensitive transcriptional activator SoxR	codon=46	CTG>GTG	L>V
MSMEG_5589	YP_889824.1	manganese transporter MntH	codon=344	GCC>GAC	A>D
MSMEG_5595	YP_889830.1	MarR family transcriptional regulator	Intergenic mutation		
MSMEG_5713	YP_889945.1	major facilitator family protein transporter	codon=278	GGT>GTT	G>V
MSMEG_5876	YP_890101.1	H-N-H endonuclease F-TflIV	codon=107	GCC>TCC	A>S
MSMEG_6646	YP_890855.1	methylisocitrate lyase	codon=31	CCG>TCG	P>S
MSMEG_6708	YP_890916.1	epoxide hydrolase	codon=217	GCC>TCC	A>S
MSMEG_6947	YP_891139.1	chromosomal replication initiation protein	codon=232	AAG>AAT	K>N

* All Functionally annotated by BLAST search.

Table S4. Results of Dunnett's multiple comparison test for in vivo testing of lcta-3368.

Dunnett's multiple comparisons test	Mean Diff.	95.00% CI of diff.	Significant?	Summary	Adjusted P Value
RIF 10 vs. Solvent control	-0.2508	-0.4126 to -0.08905	Yes	***	0.0007
RIF 10 vs. LCTA 3368 30	-0.3010	-0.4628 to -0.1392	Yes	****	<0.0001
RIF 10 vs. LCTA 3368 100	-0.2463	-0.4081 to -0.08452	Yes	***	0.0009
RIF 10 vs. LCTA 3368 100 (I.P)	-0.2194	-0.3812 to -0.05765	Yes	**	0.0037
RIF 10 vs. LCTA 3368 200	-0.2899	-0.4517 to -0.1281	Yes	****	<0.0001

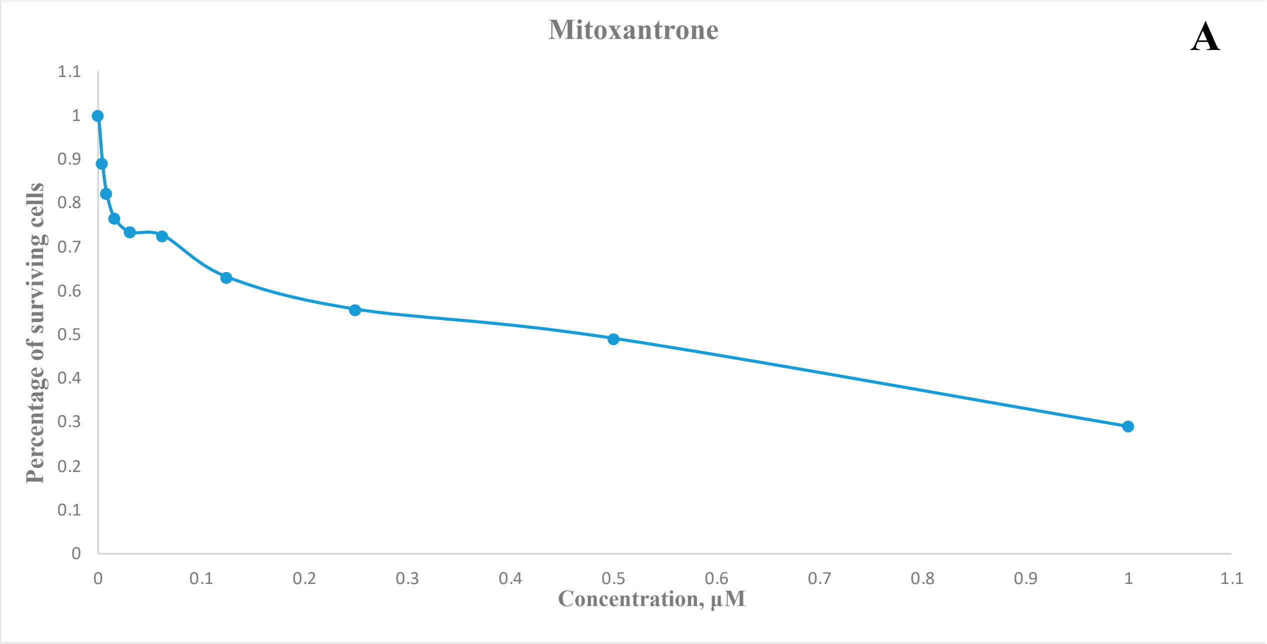
Mitoxantrone
A
Percentage of surviving cells
1.1
1
0.9
0.8
0.7
0.6
0.5
0.4
0.3
0.2
0.1
0
0
0.1
0.2
0.3
0.4
0.5
0.6
0.7
0.8
0.9
1
1.1
Concentration, µM

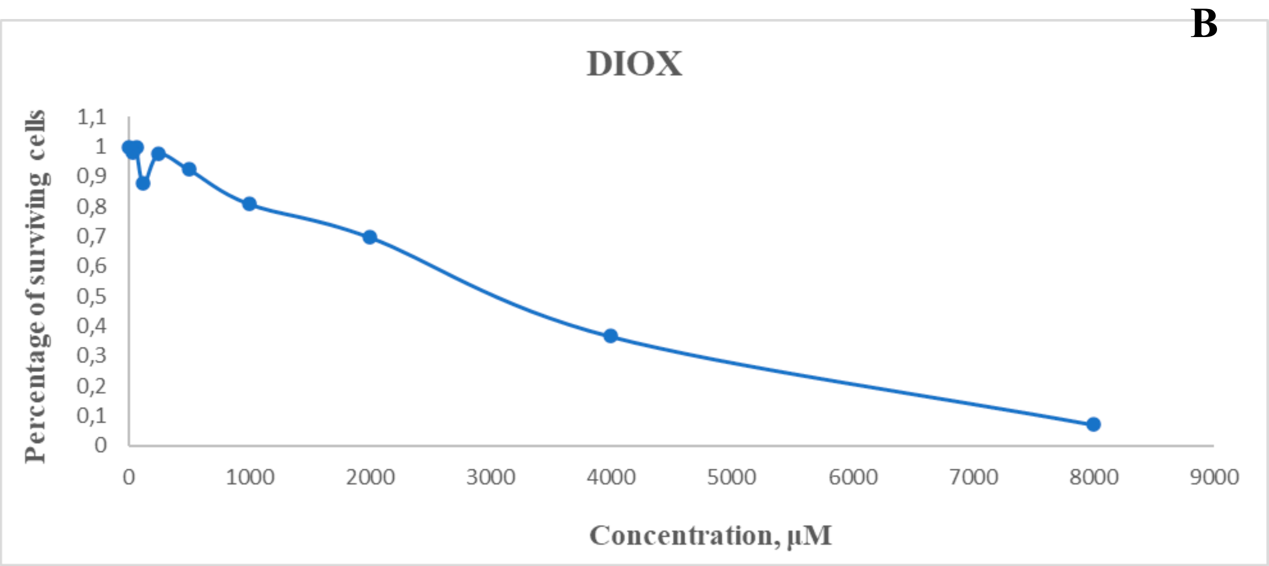
B
DIOX
Percentage of surviving cells
1,1
1
0,9
0,8
0,7
0,6
0,5
0,4
0,3
0,2
0,1
0
0
1000
2000
3000
4000
5000
6000
7000
8000
9000
Concentration, µM

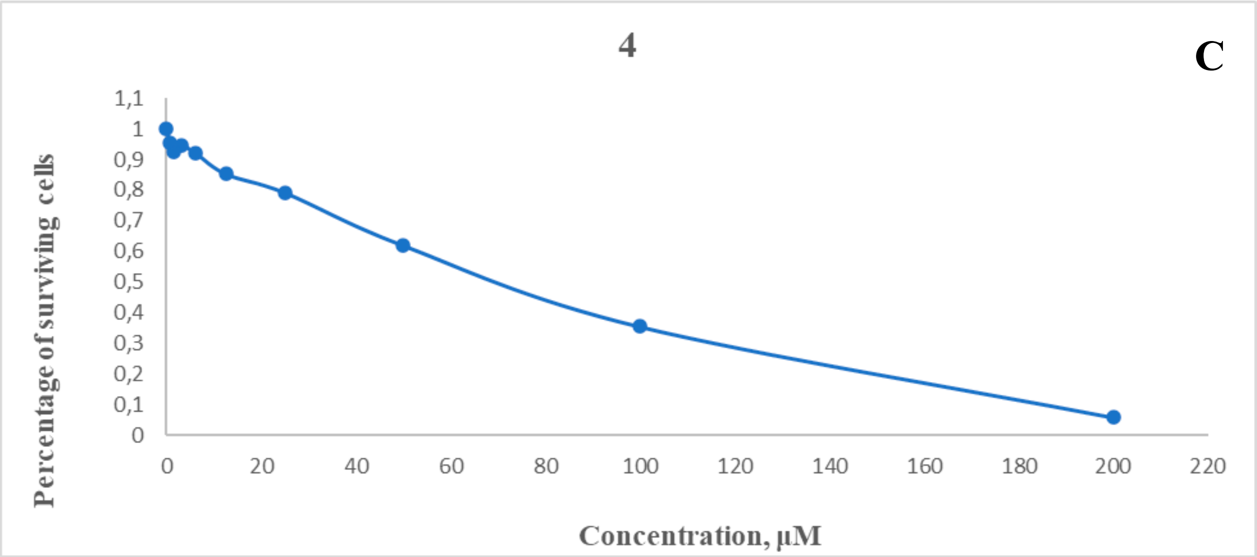

Figure S40. Results of MTT test of compounds on human fibroblasts. (A) Mitoxantrone was used as control. (B) Dioxidine (C) Tested 4.
